# Prognostic Value of Genomic Instability of m^6^A-Related lncRNAs in Lung Adenocarcinoma

**DOI:** 10.3389/fcell.2022.707405

**Published:** 2022-03-03

**Authors:** Rui Li, Jian-Ping Li, Ting-Ting Liu, Chen Huo, Jie Yao, Xiu-Li Ji, Yi-Qing Qu

**Affiliations:** ^1^ Shandong Key Laboratory of Infectious Respiratory Diseases, Department of Pulmonary and Critical Care Medicine, Qilu Hospital, Cheeloo College of Medicine, Shandong University, Jinan, China; ^2^ Department of Pulmonary Disease, Traditional Chinese Medicine Hospital of Jinan, Jinan, China; ^3^ Shandong Key Laboratory of Infectious Respiratory Diseases, Department of Pulmonary and Critical Care Medicine, Qilu Hospital of Shandong University, Jinan, China

**Keywords:** lung adenocarcinoma, performance comparison analysis, anticancer drug sensitivity analysis, prognostic risk model, genomic instability–derived m^6^A-related lncRNA

## Abstract

**Background:** Genomic instability of N6-methyladenosine (m^6^A)–related long noncoding RNAs (lncRNAs) plays a pivotal role in the tumorigenesis of lung adenocarcinoma (LUAD). Our study identified a signature of genomic instability of m^6^A-associated lncRNA signature and revealed its prognostic role in LUAD.

**Methods:** We downloaded RNA-sequencing data and somatic mutation data for LUAD from The Cancer Genome Atlas (TCGA) and the GSE102287 dataset from the Gene Expression Omnibus (GEO) database. The “Limma” R package was used to identify a network of regulatory m^6^A-related lncRNAs. We used the Wilcoxon test method to identify genomic-instability–derived m^6^A-related lncRNAs. A competing endogenous RNA (ceRNA) network was constructed to identify the mechanism of the genomic instability of m^6^A-related lncRNAs. Univariate and multivariate Cox regression analyses were performed to construct a prognostic model for internal testing and validation of the prognostic m^6^A-related lncRNAs using the GEO dataset. Performance analysis was conducted to compare our prognostic model with the previously published lncRNA models. The CIBERSORT algorithm was used to explore the relationship of m^6^A-related lncRNAs and the immune microenvironment. Prognostic m^6^A-related lncRNAs in prognosis, the tumor microenvironment, stemness scores, and anticancer drug sensitivity were analyzed to explore the role of prognostic m^6^A-related lncRNAs in LUAD.

**Results:** A total of 42 genomic instability–derived m^6^A-related lncRNAs were differentially expressed between the GS (genomic stable) and GU (genomic unstable) groups of LUAD patients. Four differentially expressed lncRNAs, 17 differentially expressed microRNAs, and 75 differentially expressed mRNAs were involved in the genomic-instability–derived m^6^A-related lncRNA-mediated ceRNA network. A prediction model based on 17 prognostic m^6^A-associated lncRNAs was constructed based on three TCGA datasets (all, training, and testing) and validated in the GSE102287 dataset. Performance comparison analysis showed that our prediction model (area under the curve [AUC] = 0.746) could better predict the survival of LUAD patients than the previously published lncRNA models (AUC = 0.577, AUC = 0.681). Prognostic m^6^A-related-lncRNAs have pivotal roles in the tumor microenvironment, stemness scores, and anticancer drug sensitivity of LUAD.

**Conclusion:** A signature of genomic instability of m^6^A-associated lncRNAs to predict the survival of LUAD patients was validated. The prognostic, immune microenvironment and anticancer drug sensitivity analysis shed new light on the potential novel therapeutic targets in LUAD.

## Introduction

Lung cancer is the most frequently diagnosed cancer and the leading cause of cancer-related deaths globally (Torre et al., 2015; Bray et al., 2018). Lung adenocarcinoma (LUAD), a type of non–small cell lung cancer (NSCLC), accounts for more than 40% of lung cancer (Jordan et al., 2017). Despite great progress in drugs, including tyrosine kinase inhibitors and immune checkpoint inhibitors, the 5-year survival rate of patients with lung adenocarcinoma was less than 15% (Zhao et al., 2018; Zhang et al., 2021). Therefore, there is an urgent need to identify new prognostic molecular biomarkers to predict survival and serve as new therapeutic targets in LUAD patients.

Genomic instability, a hallmark of cancer, results from mutations in DNA repair genes and promotes cancer development (Negrini et al., 2010; Andor et al., 2017; Seton-Rogers, 2018; Duijf et al., 2019; Hypoxic Tumors Share Geno, 2019). Recent studies have demonstrated that the characteristics of genomic instability in cancers are associated with clinical implications and prognosis (Sheffer et al., 2009; Sahin et al., 2016). Research demonstrated that human long noncoding RNA (lncRNA) LINC00657 that is induced after DNA damage maintains genomic instability by sequestering PUMILIO proteins (Lee et al., 2016). Genomic instability–related lncRNAs have a critical role in the tumorigenesis of cancers (Chua et al., 2017; Munschauer et al., 2018; Tracy et al., 2018; Petti et al., 2019; Bao et al., 2020). Recently, a study demonstrated that EZH2 mediates ribosomal DNA stability *via* silencing of lncRNA PHACTR2-AS1, which promotes breast cancer cell proliferation and metastasis (Chu et al., 2020). N^6^-methylandenosine (m^6^A)–related lncRNAs play a significant role in multiple cancers (Zhang et al., 2019a; Ni et al., 2019; Wang et al., 2019), and an m^6^A-related lncRNA was developed to predict the diagnosis and prognosis of cancers (Tu et al., 2020). Recently, a study developed mutator-derived lncRNA signatures of genome instability for predicting clinical outcomes in breast cancer (Bao et al., 2020). Although some lncRNAs have been shown to be involved in genomic instability, the specific role of genomic instability–associated m^6^A-related lncRNAs and their clinical implications in LUAD remains largely unexplored.

In this study, we identified differentially expressed m^6^A-related lncRNAs associated with genomic instability and constructed a competing endogenous RNA (ceRNA) network and then combined m^6^A-related lncRNA expression and somatic mutation profiles based on the tumor genome to reveal a prognostic m^6^A-associated lncRNA signature for predicting the survival of LUAD patients. We also further elucidated the pivotal role of prognostic m^6^A-associated lncRNA in LUAD and validated the expression of prognostic m^6^A-related lncRNAs in LUAD cells and normal bronchial epithelioid cells; meanwhile, prognostic m^6^A-related lncRNAs of the tumor microenvironment, stemness scores, anticancer drug sensitivity, and immune subtype in LUAD were also explored, providing novel therapeutic targets based on RNA modification in LUAD.

## Materials and Methods

### Data Acquisition

RNA sequencing (RNA-seq) transcription expression data, clinical characteristic data, and simple nucleotide variation (Masked Somatic Mutation from VarScan2 Variant Aggregation and Masking) data of lung adenocarcinoma (LUAD) patients were obtained from The Cancer Genome Atlas (TCGA) (https://portal.gdc.cancer.gov/). Correlation analysis was conducted to identify the m^6^A-associated lncRNAs in LUAD according to the following criteria: absolute value of Pearson’s coefficient >0.4, *p*-value < 0.001. The Gene Expression Omnibus (GEO) dataset GSE102287 was downloaded and used to validate the prognostic mutator-derived m^6^A-associated lncRNAs related to genomic instability.

### Identification of Genomic Instability–Associated m^6^A-Related lncRNAs

We used the Perl language to calculate mutation counts in LUAD patients. Patients were classified into two groups comprising the top 25% (genomic unstable [GU] group) and the bottom 25% (genomic stable [GS] group) with respect to mutation frequency. The Wilcoxon test method was used to perform the differential expression analysis of genomic instability–associated m^6^A-related lncRNAs between the GS and GU groups. Furthermore, we explored the differential expression levels of m^6^A-associated genes and the differential somatic mutation counts between the GS and GU groups using the “Limma” and “Ggpubr” R packages. Euclidean distances and Ward’s linkage method were used for hierarchical cluster analyses.

### Construction of ceRNA Network Mediated by Mutator-Derived m^6^A-Related lncRNAs

To further explore the mechanisms of mutator-derived m^6^A-related lncRNAs, we constructed a mutator-derived, m^6^A-related, lncRNA-mediated, ceRNA network. First, we used the Wilcoxon test method to perform differential expression analysis of mutator-derived m^6^A-related lncRNAs between the GS and GU groups with thresholds of |log fold change (FC)| > 1 and *p*-value < 0.05. The MiRcode database was used to determine the relationships between microRNAs (miRNAs) and lncRNAs. The miRTarBase, MiRDB, and TargetScan databases were used to determine the relationships between miRNAs and mRNAs. Finally, we obtained a mutator-derived, m^6^A-related, lncRNA-mediated, ceRNA network. We used Cytoscape 3.6.1 to visualize the genomic instability of the lncRNA–miRNA–mRNA–ceRNA network.

### 7Functional Enrichment and ConsensusPathDB Pathway Analysis

To further explore the functions of genome instability–related m^6^A-associated lncRNAs, we conducted Gene Ontology (GO) and ConsensusPathDB analysis. We used the Database for Annotation, Visualization, and Integrated Discovery to determine the functional enrichment of mutator-derived m^6^A-associated lncRNAs. Then, we used the “prepareplot.pl” package to visualize the biological processes and molecular functions of genome instability–related m^6^A-associated lncRNAs. The ConsensusPathDB human database (http://cpdb.molgen.mpg.de/) integrates interaction networks in *Homo sapiens* and currently incorporates genetic, signaling, and gene-regulatory interactions from 32 public resources (Kamburov et al., 2013). We used ConsensusPathDB to analyze the pathway enrichment of mutator-derived m^6^A-related lncRNAs.

### Construction and Validation of a Risk Prediction Model of Mutator-Derived m^6^A-Related lncRNAs

We used the “survival,” “caret,” “glmnet,” “survminer,” and “timeROC” packages to construct the prediction model of m^6^A-related lncRNAs. First, TCGA data grouping is cycled 1 time, and the groups were determined with significant training and testing at a ratio of 7:3. Then, we conducted univariate Cox regression analysis to identify the significant m^6^A-associated lncRNAs using the criterion of Cox *p*-value less than 0.05. Finally, multivariate Cox regression analysis was performed to establish the Cox prediction model. Receiver operating characteristic (ROC) curves were constructed to validate the effects of the m^6^A-related lncRNA signature on clinical outcomes. The area under the ROC curve (AUC) values were calculated to assess the ability of the prognostic m^6^A-associated lncRNAs to predict the survival of LUAD patients. We used time-dependent ROC curves to assess the performance of the m^6^A-related lncRNA signature. All statistical analyses were performed using R version 4.0.3.

### Expression, Clinical Characteristics, Immune Microenvironment, and Anticancer Drug Sensitivity Analyses of Prognostic m^6^A-Associated lncRNAs

To investigate the role of prognostic m^6^A-associated lncRNAs in LUAD, we performed correlation analyses involving expression levels of m^6^A-related lncRNAs, LUAD immune environment, stemness scores based on RNA methylation and DNA expression (RNAss and, DNAss, respectively), and drug sensitivity. We downloaded LUAD transcription expression data, RNAss and DNAss data, and TCGA LUAD phenotype-immune subtype data from UCSC-Xena (https://xenabrowser.net/). Then, we combined the expression profiles of 17 prognostic m^6^A-related lncRNAs in LUAD with the LUAD phenotype-immune subtype data, transcription expression data, and stemness scores to perform Spearman’s correlation analysis for immune subtype and stemness score. Using RNA-seq data and compound activity (DTP NCI-60) data from the CellMiner database, combined with FDA (Food and Drug Administration)-approved anticancer drugs and expression of 16 prognostic m^6^A-related lncRNAs, we performed Pearson’s correlation analysis of anticancer drug sensitivity in LUAD. All statistical analyses used R software version 4.0.3.

### Validation of Expression of 17 Prognostic m^6^A-Associated lncRNAs

To further explore the expression of 17 prognostic m^6^A-associated lncRNAs between LUAD and non-LUAD tissues, we used the paired sample t-test to perform differential expression analysis in 57 paired LUAD and adjacent non-LUAD tissues from TCGA database. Meanwhile, we used the Wilcoxon rank sum test to perform the differential expression analysis in 535 LUAD tissues and 59 adjacent non-LUAD tissues from TCGA database.

### Immune Microenvironment Analysis of Prognostic m^6^A-Associated lncRNAs

To verify the relationship of the m^6^A-associated lncRNAs and immune microenvironment, we used the CIBERSORT (Cell Type Identification by Estimating Relative Subsets of RNA Transcripts) algorithm to obtain the fraction of 22 immune cell types from six microarray public datasets based on the expression file. The Perm was set as 1,000. According to the median expression of 17 prognostic m^6^A-associated lncRNAs, we divided TCGA-LUAD patients into the high-expression group and low-expression group. A *p*-value < 0.05 was selected for further analysis in CIBERSORT results. The Mann–Whitney U test was used to compare differences in immune subtypes in high-expression and low-expression groups.

### Cell Culture

Beas-2B (human bronchial epithelioid cells) was purchased from Saibai Kang Biotechnology Co., Ltd. Human LUAD cells A549, H1299, and H1975 were purchased from Procell Life Sciences& Technology Co., Ltd. We used the short tandem repeat (STR) method to identify these cells. Beas-2B was cultivated in DMEM (PM150210) with 10% fetal bovine serum (Lonsera, Shanghai Shuangru Biotechnology Co., Ltd.) and penicillin/streptomycin (100 mg/ml), and A549, H1299, and H1975 were cultivated in RPMI-1640 medium (Gibco, Invitrogen, Carlsbad CA, United States ) with 10% fetal bovine serum (Lonsera, Shanghai Shuangru Biotechnology Co., Ltd.) and penicillin/streptomycin (100 mg/ml). All cells were cultured at 37°C in a sterile humid incubator containing 5% carbon dioxide.

### RNA Isolation, Reverse Transcription, and Quantitative Real-Time Polymerase Chain Reaction (qRT-PCR)

Total RNA was isolated from A549, H1299, H1975, and Beas-2B using TRIzol (Invitrogen, Carlsbad, CA, United States ). Then, RNA was reverse-transcribed onto cDNA using the Evo M-MLV RT Kit (Accurate Biotechnology (Hunan) Co., Ltd.) and subsequently subjected to qRT-PCR using the SYBR Green Premix Pro Taq HS qPCR Kit (Accurate Biology) according to the manufacturer’s instructions. We used GAPDH as the reference gene for 17 prognostic m^6^A-related lncRNAs. The primer sequences were synthesized by BioSune Co., Ltd. (Shanghai, China) as detailed in Supplementary Table S1. The value of the relative expression was calculated by the 2^−ΔΔCT^ method.

### Statistical Analysis

Each experiment was repeated at least thrice. All data were analyzed using GraphPad Prism 7.0 and R software. Significant differences between A549, H1299, H1975, and Beas-2B cell were evaluated by one-way ANOVA. *P*-value less than 0.05 was considered as statistically significant.

## Results

### Identification of m^6^A-Associated lncRNAs in LUAD Patients

**FIGURE 1:**
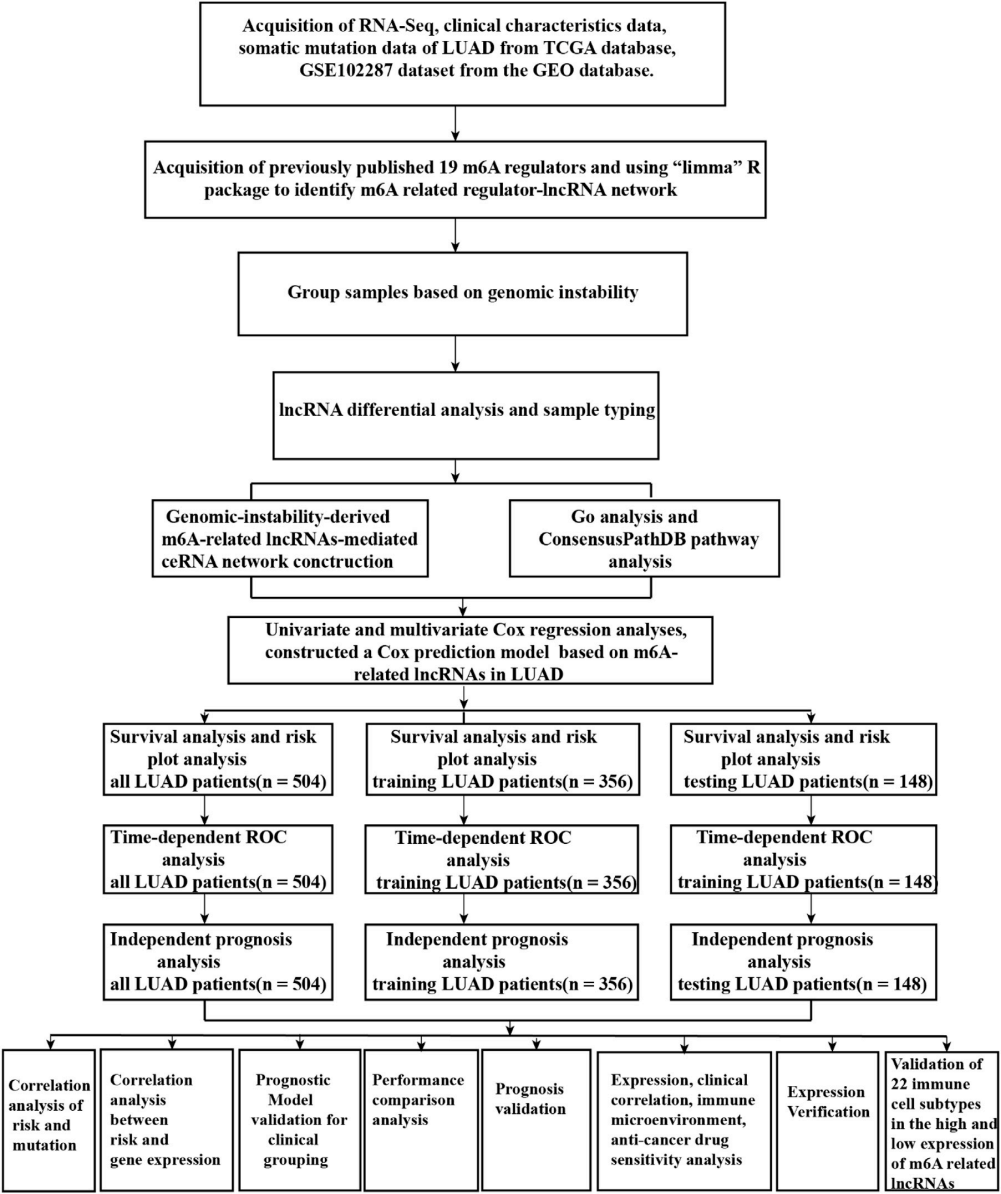
Flow chart of the whole study.

**FIGURE 2:**
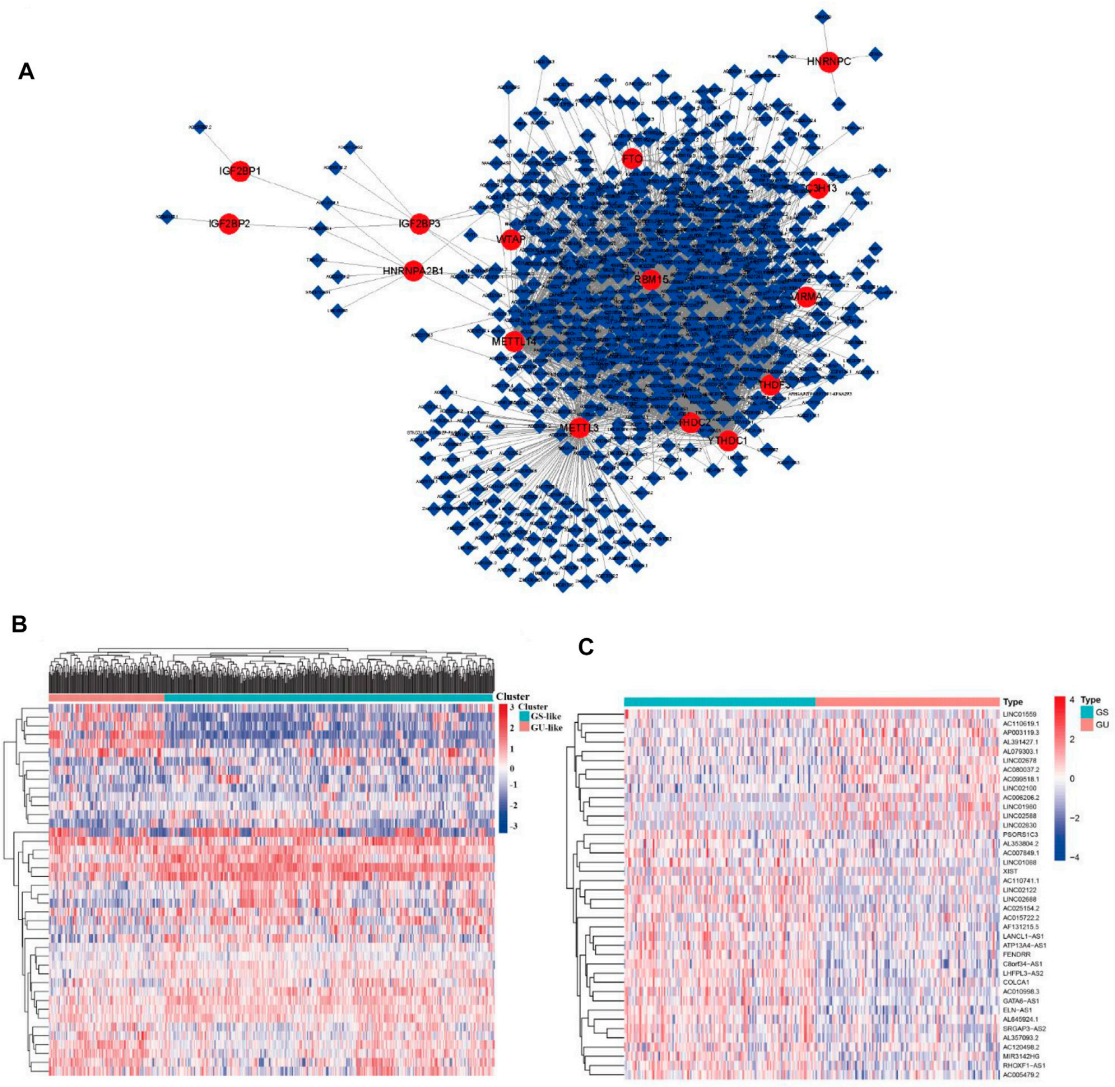
m6A regulator–lncRNA coexpression network construction, differential analysis, and hierarchical cluster analyses in LUAD. **(A)** Coexpression network between m^6^A regulators and m^6^A-related lncRNAs. Red circles indicate m6A regulators; blue diamonds indicate m^6^A-related lncRNAs. **(B)** Heatmap showing the top 20 upregulated and top 20 downregulated differentially expressed m^6^A-related lncRNAs between the GS-like group and GU-like group. **(C)** Hierarchical cluster analyses based on the expression of differentially expressed m^6^A-related lncRNAs.

A flow diagram of the whole study is shown in Figure 1. We downloaded RNA-seq transcription expression data for TCGA-LUAD patients. Then, we extracted the expression of 19 m^6^A-related regulators (METTL3, VIRMA, METTL14, ZC3H13, RBM15, RBM15B, WTAP, HNRNPC, YTHDC1, HNRNPA2B1, YTHDC2, IGF2BP1, YTHDF1, IGF2BP2, YTHDF2, IGF2BP3, YTHDF3, ALKBH5, and FTO) from The Cancer Genome Atlas (TCGA) (https://portal.gdc.cancer.gov/). Next, according to the standard filters of *p* < 0.001 and correlation coefficient >0.4, 2,710 correlated pairs of m^6^A-related regulators and m^6^A-related lncRNA correlations were identified (Supplementary Table S2) (Figure 2A).

### Identification of Genome Instability–Derived m^6^A-Related lncRNAs in LUAD Patients

**FIGURE 3:**
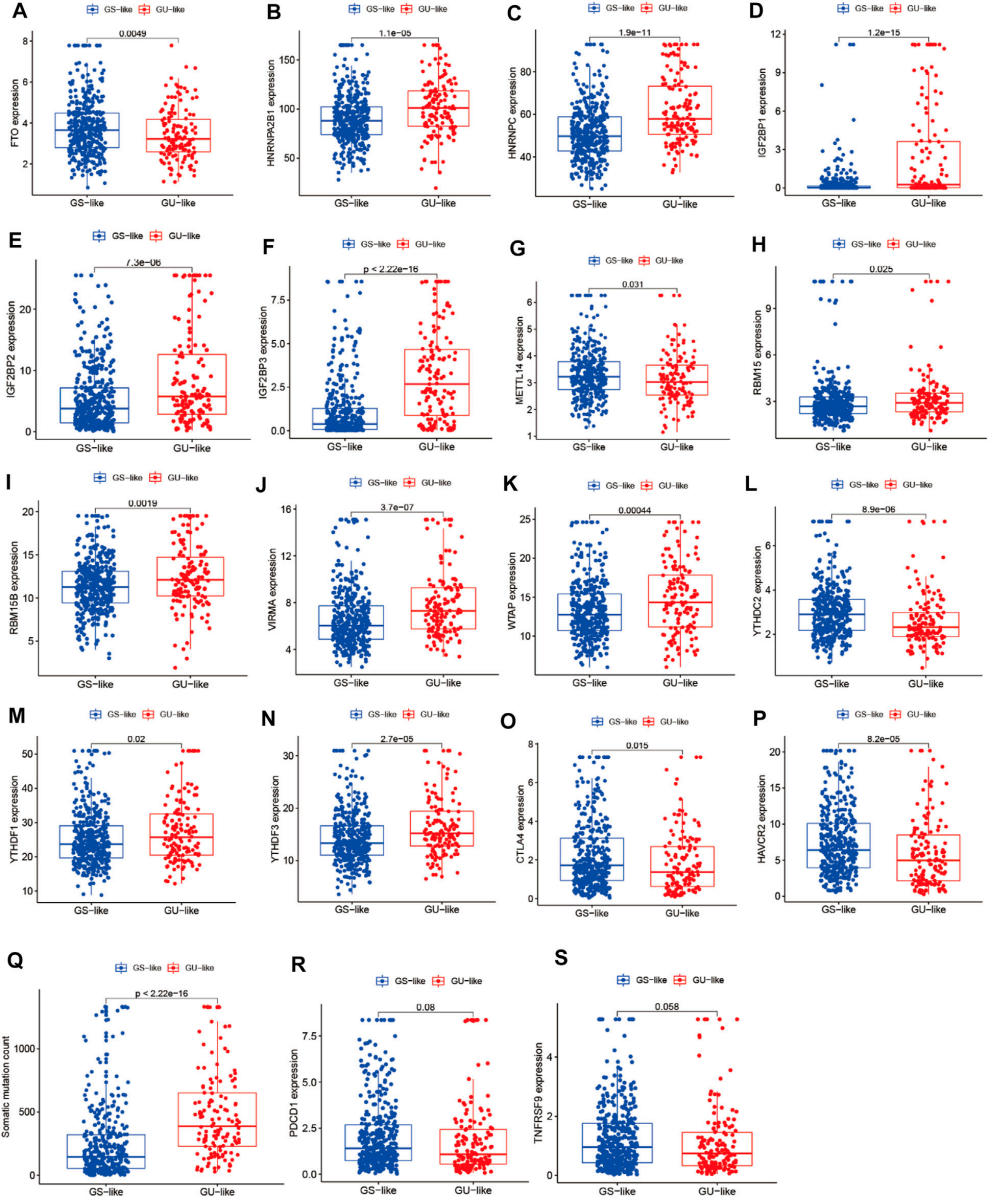
Correlation analysis of LUAD clusters, expression of m^6^A regulators, CTLA4, HAVCR2, PDCD1, 4-1BB (TNFRSF9), and somatic mutation counts of LUAD. **(A–N)** Correlation analysis of LUAD clusters and expression of m^6^A regulators. **(O–S)** Correlation analysis of LUAD clusters and expression of CTLA4, HAVCR2, PDCD1, and 4-1BB (TNFRSF9). **(Q)** Correlation analysis of LUAD clusters and somatic mutation counts.

To identify genome instability–derived m^6^A-related lncRNAs, we first downloaded TCGA somatic mutation data from the GDC Data Portal. Then, we calculated the cumulative number of somatic mutations per LUAD patient and sorted patients in descending order based on this number; the lowest 25% (n = 134) and the top 25% (n = 139) patients were defined as the GS-like group and GU-like group, respectively (Figure 2B). Next, 42 genome instability–derived m^6^A-related lncRNAs were differentially expressed between the GS and GU groups based on |logFC|≥1 and false discovery rate adjusted *p*-value < 0.05. In total, 12 genome instability–derived m^6^A-related lncRNAs were upregulated and 30 were downregulated in the GU group (Table 1). Unsupervised hierarchical clustering analysis was performed for 535 LUAD patients from TCGA dataset using the set of 42 differentially expressed genome instability–derived m^6^A-related lncRNAs (Figure 2C). All 535 LUAD patients were clustered into two cohorts according to the expression levels of differentially expressed genome instability–derived m^6^A-related lncRNAs. The somatic mutation count was significantly different between the two cohorts. We defined the cohort with the lower cumulative somatic mutation count as the GS group and the one with the higher cumulative somatic mutation count as the GU group. Then, we analyzed the differential expression of 19 m^6^A-related regulators between the GU and GS groups. As shown in Figure 3, the expression levels of FTO, HNRNPC, HNRNPA2B1, IGF2BP3, IGF2BP1, IGF2BP2, YTHDC2, YTHDF1, YTHDF3, METTL14, RBM15, VIRMA, RBM15B, and WTAP differed significantly between GU and GS patients (*p* < 0.05). To explore the role of immune checkpoint molecules, we performed differential expression analysis for CTLA4 (T-lymphocyte–associated protein 4), HAVCR2 (hepatitis A virus cellular receptor 2), PDCD1, and 4-1 BB (TNFRSF9) between the two groups. CTLA4 showed higher expression in the GU group than in the GS group (*p* = 0.015), whereas the reverse was true for HAVCR2 (*p* = 8.2e-05). However, PDCD1 and 4-1 BB (TNFRSF9) had no significant difference between the two groups (*p* = 0.08, *p* = 0.058). Furthermore, the somatic mutation counts of LUAD patients showed significant differences between the two groups (*p* < 2.22e-16).

**TABLE 1:** Differentially expressed genomic instability–derived m^6^A-related lncRNAs in the GS-like cohort and GU-like cohort.

Lnc	conMean	treatMean	logFC	p-value	Fdr
AL391427.1	0.739627	2.1293128	1.525518	0.015829	0.032507
AC120498.2	1.597165	0.6001846	−1.41203	0.000657	0.00225
LINC02588	0.304,194	0.7786175	1.355923	5.62E-05	0.000305
COLCA1	4.458726	1.9803476	−1.17088	7.25E-10	2.15E-08
GATA6-AS1	1.080457	0.2267509	−2.25246	6.26E-12	3.40E-10
AC015722.2	0.599252	0.2992237	−1.00194	0.025531	0.049174
AL357093.2	3.143296	0.9129523	−1.78367	3.25E-07	3.78E-06
LHFPL3-AS2	5.168442	0.9177162	−2.49361	3.12E-16	2.03E-13
AP003119.3	0.442823	1.205654	1.445013	1.43E-05	9.52E-05
PSORS1C3	2.804098	1.2591726	−1.15506	4.83E-06	3.93E-05
ATP13A4-AS1	2.102016	0.57317	−1.87474	1.65E-06	1.53E-05
ELN-AS1	5.689761	2.3339656	−1.28558	1.25E-14	2.72E-12
AL645924.1	1.604076	0.6361313	−1.33435	6.70E-06	5.17E-05
LINC02100	0.590293	1.1924707	1.01445	0.000316	0.001254
BX640514.2	1.348153	0.622749	−1.11426	6.03E-12	3.40E-10
AC110619.1	0.409055	0.9909137	1.276466	0.008391	0.020083
LINC02678	0.483487	1.0305523	1.091869	5.28E-05	0.000289
AC007849.1	1.67354	0.8269208	−1.01708	0.014723	0.030872
SRGAP3-AS2	3.412214	0.9639636	−1.82366	4.05E-08	7.98E-07
AC025154.2	2.884158	0.8866555	−1.7017	2.33E-13	3.79E-11
LINC02688	1.30119	0.3034674	−2.10022	4.22E-10	1.41E-08
FENDRR	1.118096	0.3577013	−1.64422	4.33E-10	1.41E-08
AL079303.1	0.338798	0.7199056	1.087382	8.41E-05	0.000427
LINC01088	1.231257	0.203398	−2.59775	0.000403	0.001533
LINC02830	0.242358	0.9975995	2.041319	3.49E-05	0.000203
AC005479.2	1.704266	0.8489956	−1.00532	5.38E-08	9.90E-07
LINC01980	0.209723	1.5514401	2.887051	1.10E-07	1.70E-06
RHOXF1-AS1	2.139892	0.8688171	−1.30041	4.23E-11	1.83E-09
LANCL1-AS1	0.535622	0.2673439	−1.00252	0.000504	0.001854
AC010998.3	1.444093	0.2417359	−2.57866	4.34E-08	8.30E-07
XIST	6.009141	2.9288823	−1.03681	0.00272	0.007941
LINC02122	0.918007	0.2753383	−1.7373	1.18E-08	2.75E-07
AC099518.1	0.362166	0.9796389	1.4356	4.81E-11	1.96E-09
AL590226.1	0.688495	0.3137933	−1.13363	4.57E-07	4.88E-06
AC006206.2	0.366075	0.8736432	1.254904	8.64E-06	6.46E-05
AF131215.5	1.445376	0.7210449	−1.00328	1.45E-07	2.15E-06
MIR3142HG	1.333473	0.6487218	−1.03952	9.53E-08	1.51E-06
AC080037.2	1.289528	2.9810986	1.209001	8.83E-06	6.46E-05
AL353804.2	1.228319	0.5766532	−1.09091	0.004716	0.012739
AC110741.1	1.609459	0.2224454	−2.85505	9.66E-06	6.91E-05
LINC01559	1.244751	0.4687693	−1.40891	0.009449	0.022048
C8orf34-AS1	4.808459	1.699385	−1.50056	1.96E-15	6.37E-13

### CeRNA Network Construction

**FIGURE 4:**
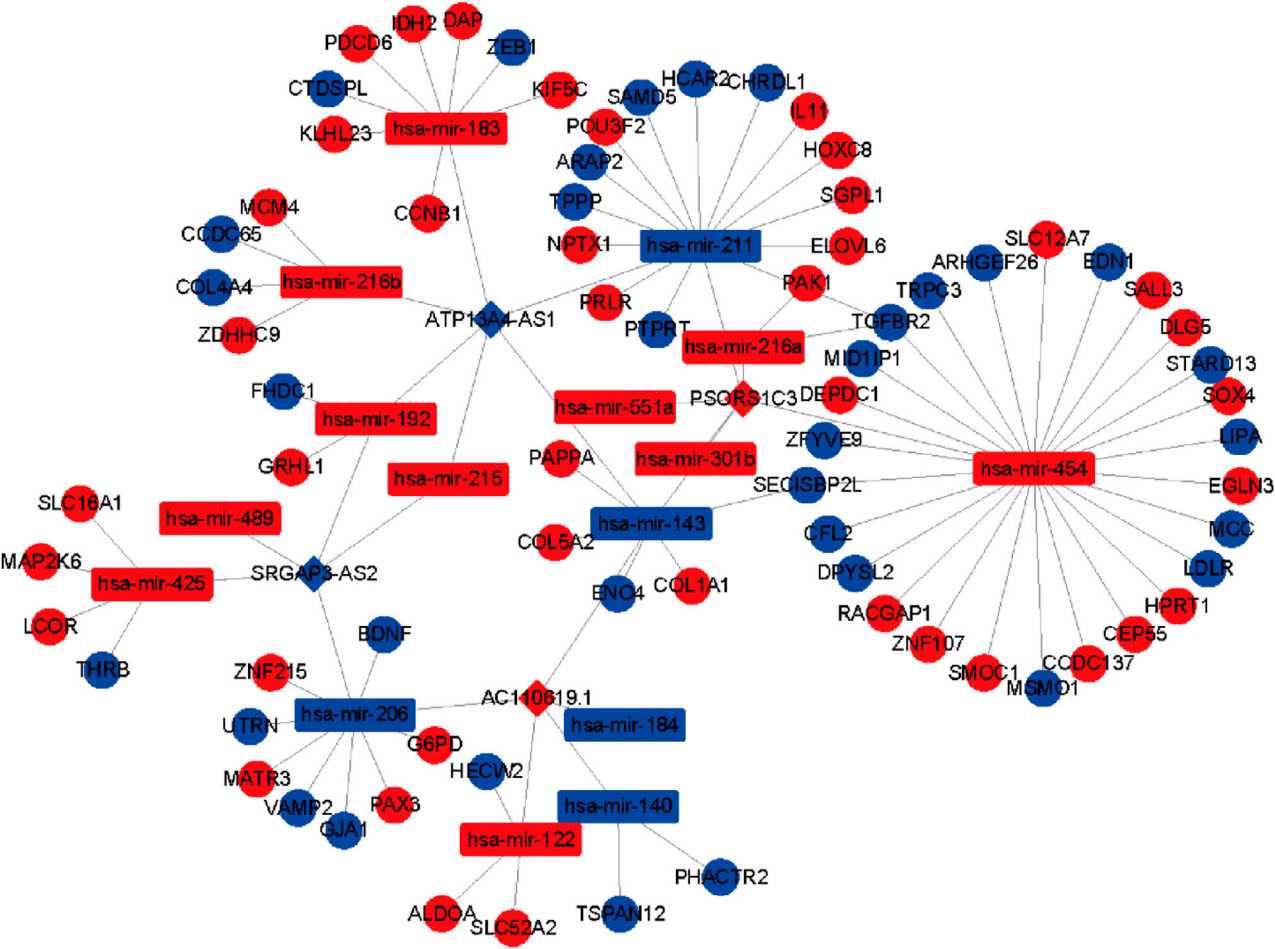
Construction of the ceRNA network. Red and blue circles indicate upregulated and downregulated mRNAs, respectively. Red and blue rectangles indicate upregulated and downregulated miRNAs, respectively. Red and blue diamonds indicate upregulated and downregulated genomic instability–derived differentially expressed m^6^A-related lncRNAs, respectively.

To further identify the mechanism of genome instability–associated m^6^A-related lncRNAs in LUAD patients, we established the genomic instability of the lncRNA-mediated ceRNA network. First, we searched for differentially expressed genome instability–derived m^6^A-related lncRNAs in the miRcode database and presented 306 pairs of interacting miRNAs and lncRNAs using Perl language. Then, we identified 17 of 298 identified differentially expressed miRNAs (DEmiRNAs) interacting with four differentially expressed genome instability–derived m^6^A-related lncRNAs. The interactions between differentially expressed miRNAs and lncRNAs are shown in Table 2. Next, we identified 17 DEmiRNAs targeting 436mRNAs using TargetScan ((http://www.targetscan.org/), miRTarbase ((http://mirtarbase.mbc.nctu.edu.tw/), and miRDB (http://www.mirdb.org/). The correlations between mRNAs and miRNAs are shown in Table 3. Finally, 75 mRNAs were used to construct the network of genome instability–derived m^6^A-related lncRNAs. Furthermore, we constructed a network of genome instability–derived m^6^A-related lncRNA-mediated ceRNA network incorporating four differentially expressed lncRNAs (DElncRNAs), 17DEmiRNAs, and 75 differentially expressed mRNAs (Figure 4).

**TABLE 2:** Interaction between differentially expressed m^6^A-related lncRNAs and miRNAs associated with genomic instability.

lncRNA	miRNA					
PSORS1C3	hsa-mir-551a	hsa-mir-301b	hsa-mir-454	hsa-mir-143	hsa-mir-211	hsa-mir-216a
ATP13A4-AS1	hsa-mir-143	hsa-mir-183	hsa-mir-192	hsa-mir-215	hsa-mir-211	hsa-mir-216b
SRGAP3-AS2	hsa-mir-192	hsa-mir-215	hsa-mir-206	hsa-mir-425	hsa-mir-489	
AC110619.1	hsa-mir-140	hsa-mir-143	hsa-mir-184	hsa-mir-206	hsa-mir-122	

**TABLE 3:** Interaction between differentially expressed miRNAs and mRNAs associated with genomic instability.

miRNA	mRNA					
hsa-mir-454	STARD13	EDN1	DPYSL2	RACGAP1	TRPC3	ZFYVE9
	SLC12A7	ARHGEF26	MSMO1	LIPA	CCDC137	
	SOX4	TGFBR2	MID1IP1	CEP55	ZNF107	
	DEPDC1	CFL2	HPRT1	DLG5	EGLN3	
	SALL3	LDLR	SECISBP2L	SMOC1	MCC	
hsa-mir-211	TPPP	HCAR2	ELOVL6	SGPL1	TGFBR2	
	CHRDL1	NPTX1	HOXC8	SAMD5	PRLR	
	POU3F2	IL11	PTPRT	ARAP2		
hsa-mir-122	ALDOA					
	SLC52A2					
	HECW2					
hsa-mir-216a	PAK1					
	TGFBR2					
hsa-mir-143	COL5A2	PAPPA				
	ENO4	COL1A1				
hsa-mir-183	PDCD6	DAP	CTDSPL			
	KIF5C	ZEB1	CCNB1			
	KLHL23	IDH2				
hsa-mir-206	UTRN	ZNF215				
	G6PD	VAMP2				
	PAX3	GJA1				
	BDNF	MATR3				
hsa-mir-425	THRB	SLC16A1				
	LCOR	MAP2K6				
hsa-mir-192	GRHL1					
	FHDC1					
hsa-mir-216b	COL4A4	MCM4				
	CCDC65	ZDHHC9				
hsa-mir-140	TSPAN12					

### Functional and ConsensusPathDB Analysis of Genome Instability–Derived m^6^A-Related lncRNAs

**FIGURE 5:**
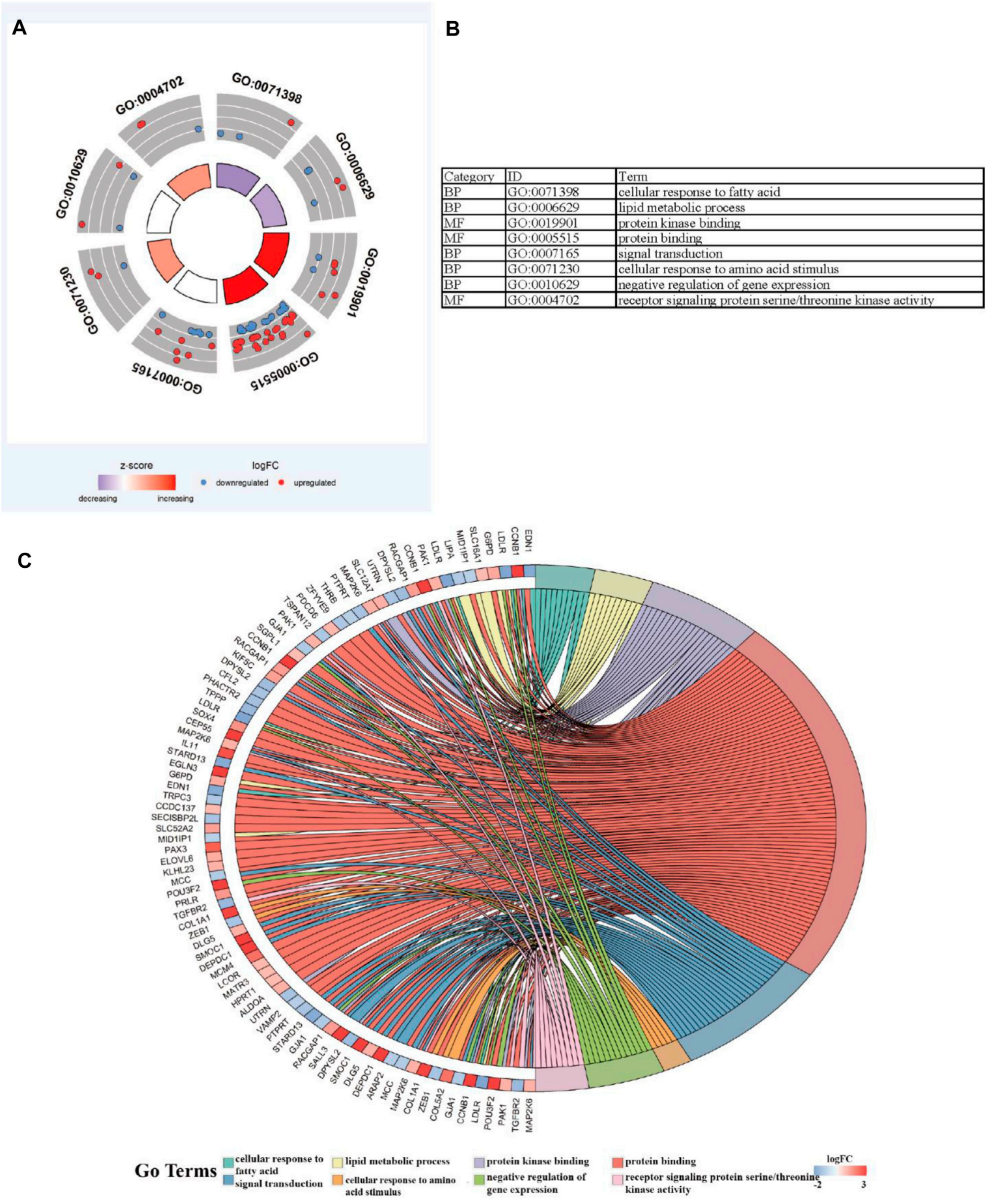
Functional enrichment analysis. **(A–C)** GO analysis indicating top eight GO terms for which differentially expressed mRNAs were most enriched.

**FIGURE 6:**
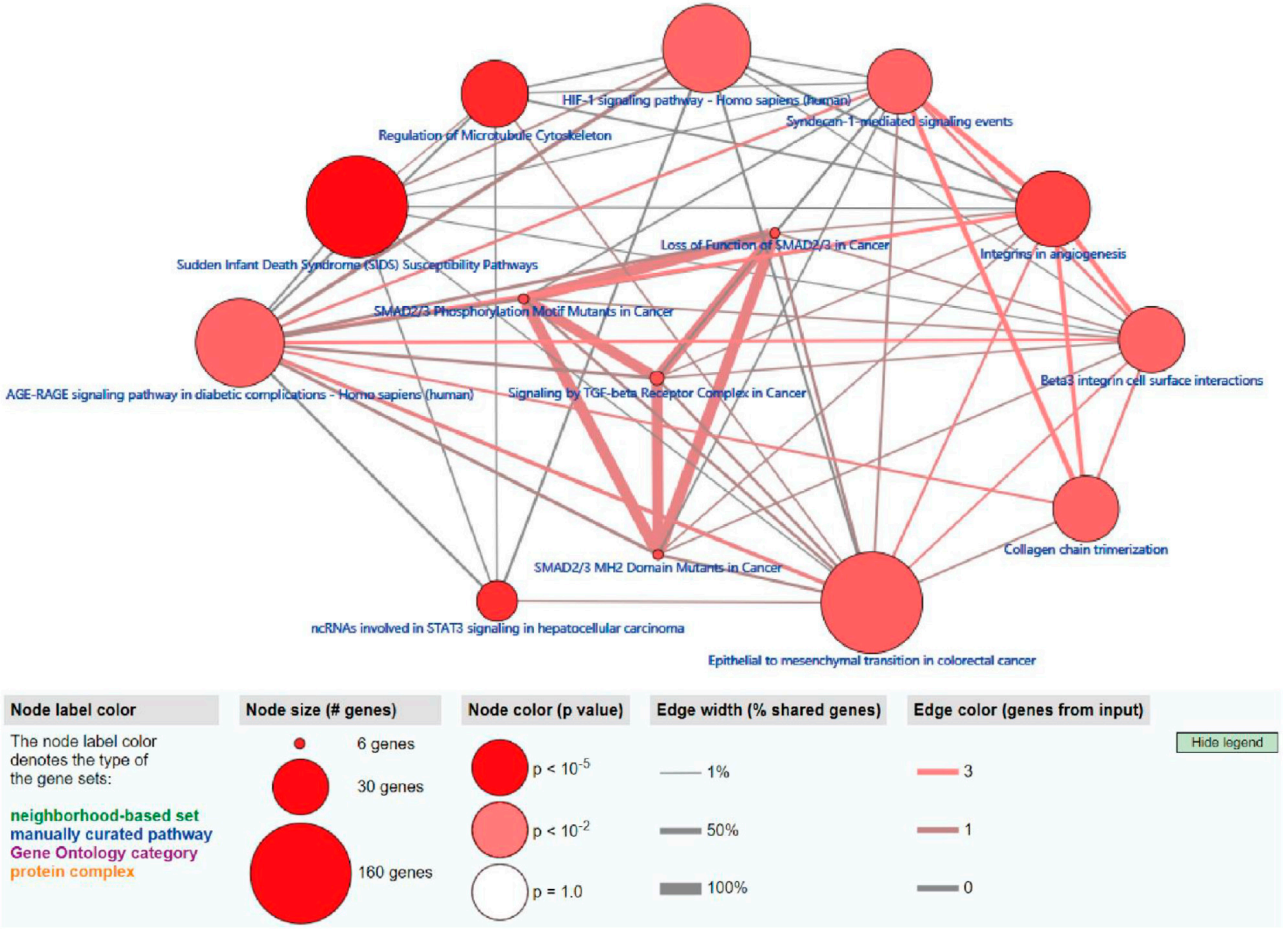
ConsensusPathDB analysis indicating that differentially expressed mRNAs were most enriched in 14 pathways.

To further explore the potential functions and pathways involving the genome instability–derived m^6^A-related lncRNAs, we conducted functional enrichment and ConsensusPathDB pathway analyses. According to the genome instability–derived m^6^A-related lncRNA-mediated ceRNA network, we obtained 75 lncRNA-correlated mRNAs. As shown in Figure 5, the 75 mRNAs were most enriched in eight GO terms: the cellular response to fatty acids, lipid metabolic process, protein kinase binding, protein binding, signal transduction, cellular response to amino acid stimulus, negative regulation of gene expression, and receptor signaling protein serine/threonine kinase activity. The ConsensusPathDB pathway analyses demonstrated that 75 mRNAs were most enriched in 14 pathways: sudden infant death syndrome (SIDS) susceptibility pathways, regulation of the microtubule cytoskeleton, ncRNAs involved in STAT3 signaling in hepatocellular carcinoma, integrins in angiogenesis, SMAD2/3 phosphorylation motif mutants in cancer, SMAD2/3 MH2 domain mutants in cancer, loss of function of SMAD2/3 in cancer, signaling by TGF-beta receptor complex in cancer, epithelial–mesenchymal transition in colorectal cancer, syndecan-1–mediated signaling events, AGE-RAGE signaling pathway in diabetic complications – Homo sapiens (human), collagen chain trimerization, beta3 integrin cell surface interactions, and HIF-1 signaling pathway – Homo sapiens (human) (Figure 6) (Table 4).

**TABLE 4:** ConsensusPathDB pathway analysis.

Pathway source	Pathway name	*p*-value	q-value
Wikipathways	Sudden infant death syndrome (SIDS) susceptibility pathways	5.70E-06	0.00128
Wikipathways	Regulation of microtubule cytoskeleton	4.73E-05	0.00468
Wikipathways	ncRNAs involved in STAT3 signaling in hepatocellular carcinoma	6.23E-05	0.00468
PID	Integrins in angiogenesis	0.000164	0.00901
Reactome	SMAD2/3 phosphorylation motif mutants in cancer	0.00028	0.00901
Reactome	SMAD2/3 MH2 domain mutants in cancer	0.00028	0.00901
Reactome	Loss of function of SMAD2/3 in cancer	0.00028	0.00901
Reactome	Signaling by TGF-beta receptor complex in cancer	0.000392	0.011
Wikipathways	Epithelial–mesenchymal transition in colorectal cancer	0.000665	0.0153
PID	Syndecan-1–mediated signaling events	0.000872	0.0153
KEGG	AGE-RAGE signaling pathway in diabetic complications – Homo sapiens (human)	0.000919	0.0153
Reactome	Collagen chain trimerization	0.000933	0.0153
PID	Beta3 integrin cell surface interactions	0.000933	0.0153
KEGG	HIF-1 signaling pathway – Homo sapiens (human)	0.000955	0.0153

### Construction of the Cox Prediction Model of m^6^A-Associated lncRNAs in LUAD

**FIGURE 7:**
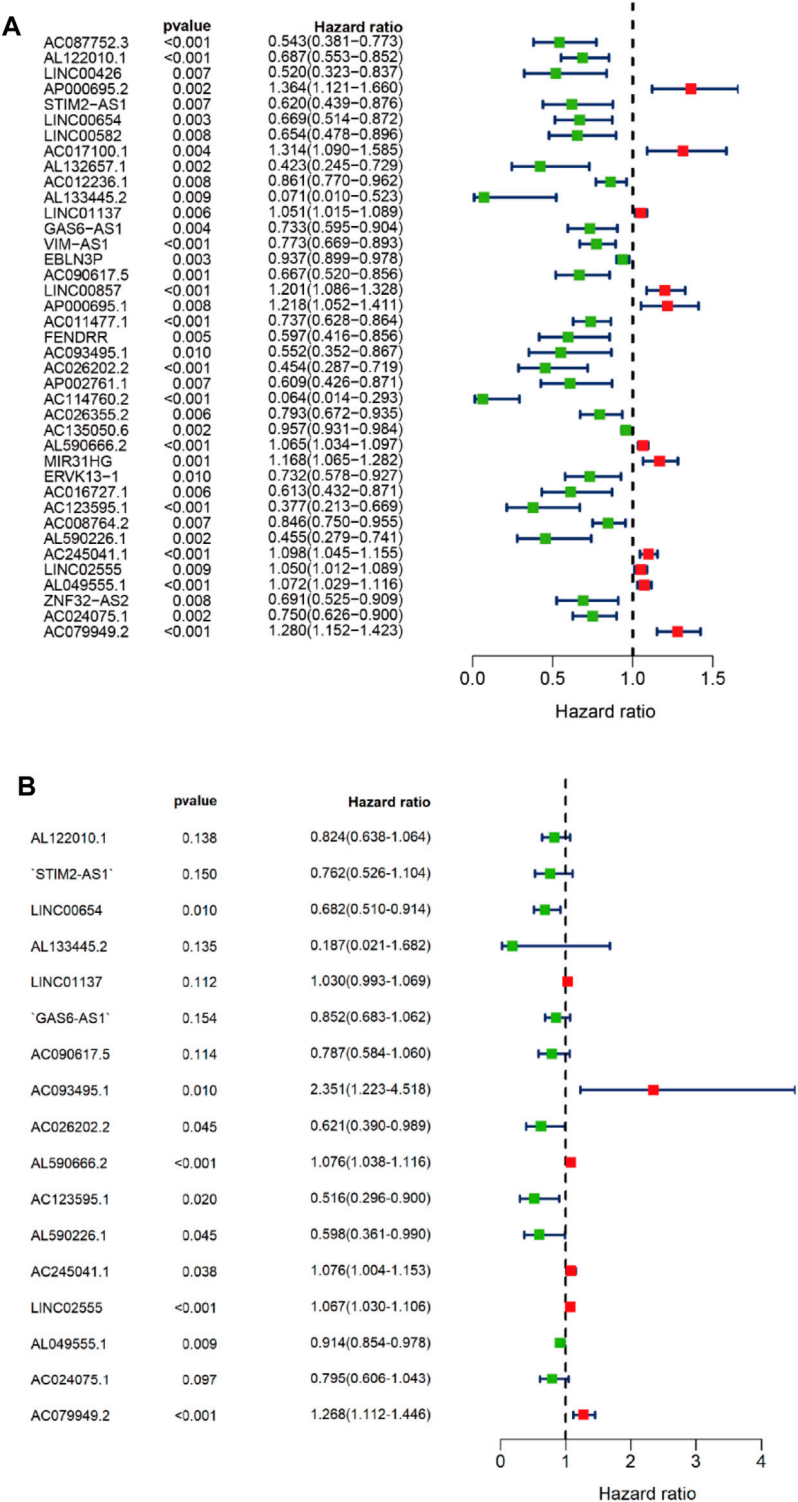
Univariate and multivariate Cox regression analyses. **(A)** Forest map of univariate Cox regression analysis. **(B)** Forest map of multivariate Cox regression analysis. Red and green circles indicate high-risk and low-risk factors, respectively.

**FIGURE 8:**
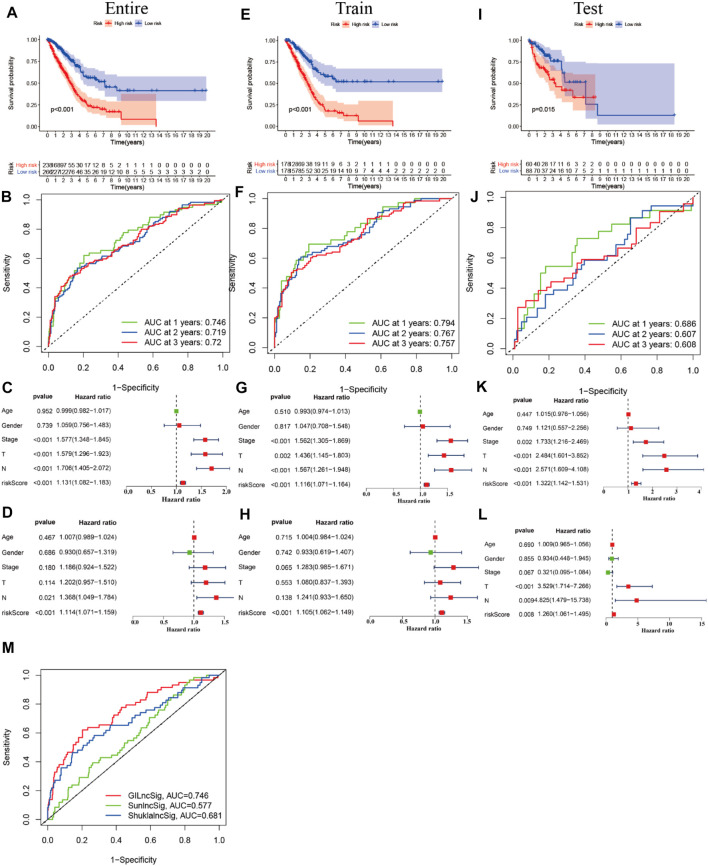
Prognostic value of m^6^A-related lncRNAs of entire, training, and testing cohorts of LUAD patients. **(A,E,I)** Survival analysis of the prognostic model of the entire, training, and testing cohorts of LUAD patients. **(B,F,J)** ROC AUC at 1, 2, and 3 years in the entire, training, and testing cohorts. **(C,G,K)** Forest map of univariate Cox independent regression analyses of the prognostic model for the entire, training, and testing cohorts. **(D,H,L)** Forest map of multivariate Cox independent regression analyses of the prognostic model for the entire, training, and testing cohorts. **(M)** ROC curve of AUC analysis for our prediction model and other published prognostic models.

First, we tried constructing a Cox prediction model of genomic instability of m^6^A-related lncRNAs in LUAD patients. However, the results showed that there was only one group in LUAD patients. Then, we further explored whether m^6^A-associated lncRNAs could serve as prognostic biomarkers for predicting the clinical outcomes of LUAD patients and established a Cox prediction model. First, we combined the expression level of m^6^A-related lncRNAs and the complete survival status and survival time data of 535 LUAD patients, Then, the patients were divided into two cohorts in the ratio of 7:3: training cohort (n = 356) and testing cohort (n = 148). Next, univariate Cox regression analysis of the training TCGA cohort showed that 39 m^6^A-related lncRNAs were significantly related to overall survival (*p* < 0.05) (Figure 7A) (Table 5). We selected these 39 prognosis-related lncRNAs for multivariate Cox regression analysis. This yielded 17 m^6^A-related lncRNAs which were used to construct the Cox prediction model (Figure 7B) (Table 6). The weighted relative coefficients in the Cox prediction model were as follows: riskscore = (-0.1938)×AL122010.1 expression + (-0.2721) × STIM2-AS1 expression + (-0.3821) × LINC00654 expression + (-1.6785) × AL133445.2 expression +0.300 × LINC01137 expression + (-0.1602) × GAS6-AS1 expression + (-0.2401) × AC090617.5 expression +0.8548 × AC093495.1 expression + (-0.4769) × AC026202.2 expression +0.0733 × AL590666.2 expression + (-0.6618) × AC123595.1 expression + (-0.5149) × AL590226.1 expression +0.0734 × AC245041.1 expression +0.0648 × LINC02555 expression + (-0.0900) × AL049555.1 expression + (-0.2295) × AC024075.1 expression +0.2374 × AC079949.2 expression). According to the median risk scores (1.62, 1.69, and 1.41), we divided our entire TCGA set, training TCGA set, and testing TCGA set into high-risk and low-risk groups. Survival analysis on these three sets, (entire, training, and testing) showed that patients in the high-risk groups had worse prognosis than those in the low-risk groups (*p* < 0.05) (Figures 8A,E,I). Time-dependent ROC curves for the entire, training, and testing sets at 1 year, 2 years, and 3 years showed that the Cox prediction model could moderately well-predict the overall survival of LUAD patients (Figures 8B,F,J). Independent prognosis analyses showed that pathological N stage and riskscore were independent prognostic factors for overall survival in the entire TCGA-LUAD cohort (*p* < 0.05) (Figure 8D). Riskscore was an independent prognostic factor for survival in TCGA training LUAD cohort (*p* < 0.05) (Figure 8H), and pathological T and N stages were independent prognostic factors for clinical outcomes in the testing TCGA-LUAD cohort (*p* < 0.05) (Figure 8L).

**TABLE 5:** Univariate Cox regression analysis of training TCGA sets of prognostic m^6^A-related lncRNAs in LUAD.

Id	HR	HR.95L	HR.95H	P-value
AC087752.3	0.542898	0.381069	0.773451	0.000718
AL122010.1	0.686524	0.553259	0.85189	0.000636
LINC00426	0.519749	0.322805	0.836849	0.007084
AP000695.2	1.36425	1.121436	1.65964	0.001896
STIM2-AS1	0.620276	0.438964	0.876479	0.006782
LINC00654	0.669227	0.513822	0.871632	0.002892
LINC00582	0.654052	0.477511	0.895861	0.008167
AC017100.1	1.313955	1.08953	1.584608	0.004273
AL132657.1	0.422515	0.244926	0.728869	0.001956
AC012236.1	0.860704	0.770124	0.961938	0.008195
AL133445.2	0.070744	0.009562	0.523,412	0.009487
LINC01137	1.0512	1.014756	1.088953	0.005543
GAS6-AS1	0.733355	0.594775	0.904222	0.003706
VIM-AS1	0.77309	0.669,141	0.893,188	0.000477
EBLN3P	0.937445	0.898916	0.977625	0.002555
AC090617.5	0.666939	0.519893	0.855575	0.001436
LINC00857	1.200585	1.085546	1.327815	0.000375
AP000695.1	1.21844	1.051947	1.411284	0.008401
AC011477.1	0.736861	0.628316	0.864157	0.000173
FENDRR	0.596549	0.41559	0.856302	0.005092
AC093495.1	0.552042	0.351596	0.866765	0.009847
AC026202.2	0.453855	0.286569	0.718797	0.000759
AP002761.1	0.60942	0.42622	0.871364	0.006632
AC114760.2	0.063983	0.013969	0.293068	0.000399
AC026355.2	0.792626	0.672035	0.934856	0.005781
AC135050.6	0.957429	0.931193	0.984404	0.002149
AL590666.2	1.065222	1.03391	1.097483	3.31E-05
MIR31HG	1.168149	1.064815	1.28151	0.001006
ERVK13-1	0.732015	0.578263	0.926648	0.009507
AC016727.1	0.61342	0.43201	0.871007	0.006295
AC123595.1	0.377159	0.212785	0.668509	0.000841
AC008764.2	0.846261	0.750203	0.954619	0.006618
AL590226.1	0.454754	0.279123	0.740897	0.001555
AC245041.1	1.098409	1.045002	1.154544	0.000223
LINC02555	1.049972	1.012015	1.089352	0.009439
AL049555.1	1.07154	1.028612	1.11626	0.000925
ZNF32-AS2	0.690664	0.524669	0.909176	0.008318
AC024075.1	0.75031	0.625778	0.899624	0.00192
AC079949.2	1.280291	1.151559	1.423413	4.88E-06

**TABLE 6:** Multivariate Cox regression analysis of m^6^A-related lncRNAs in LUAD.

Id	Coef	HR	HR.95L	HR.95H	P-value
AL122010.1	-0.19381	0.823,814	0.637,802	1.064074	0.137,723
`STIM2-AS1′	-0.27212	0.761,762	0.525,833	1.103,547	0.150,165
LINC00654	-0.38207	0.682,445	0.509,628	0.913,864	0.010331
AL133445.2	-1.67846	0.18666	0.020718	1.681,696	0.134,521
LINC01137	0.029974	1.030427	0.992,992	1.069275	0.112,405
`GAS6-AS1′	-0.16018	0.851994	0.683488	1.062044	0.154,276
AC090617.5	-0.24007	0.78657	0.583825	1.059723	0.11444
AC093495.1	0.854751	2.350789	1.223229	4.517725	0.010332
AC026202.2	-0.47686	0.62073	0.389614	0.988944	0.044776
AL590666.2	0.073257	1.076007	1.037656	1.115775	7.61E-05
AC123595.1	-0.66175	0.515946	0.295717	0.900187	0.019793
AL590226.1	-0.51491	0.597555	0.360773	0.989743	0.045499
AC245041.1	0.073357	1.076115	1.003925	1.153496	0.038404
LINC02555	0.064844	1.066992	1.02981	1.105517	0.00034
AL049555.1	-0.09004	0.91389	0.853771	0.978242	0.009499
AC024075.1	-0.22946	0.794964	0.606024	1.042809	0.097476
AC079949.2	0.237358	1.267895	1.111631	1.446125	0.000405

### Performance Comparison of the LncSig With the Signature of 17 Prognostic m^6^A-Related lncRNAs

We compared the performance of our m^6^A-related lncRNA signature with the previously published lncRNA signatures: a five-lncRNA signature from Sun’s study (SunlncSig) (Sun et al., 2020) and a four-lncRNA signature from Shukla’s study (ShuklalncSig) (Shukla et al., 2017). We extracted the published lncRNA expression and combined it with our risk profile and complete survival information for the performance comparison analysis. As shown in Figure 8M, the AUC for overall survival for the signature of 17 m^6^A-related lncRNAs was 0.746, significantly higher than that for SunlncSig (AUC = 0.577) and ShuklalncSig (AUC = 0.681). Performance comparison analysis showed that our signature could better predict the clinical outcomes of LUAD patients than the two recently published lncRNA signatures.

### Correlation Analysis of the Expression of m^6^A Regulators and the Risk Model

**FIGURE 9:**
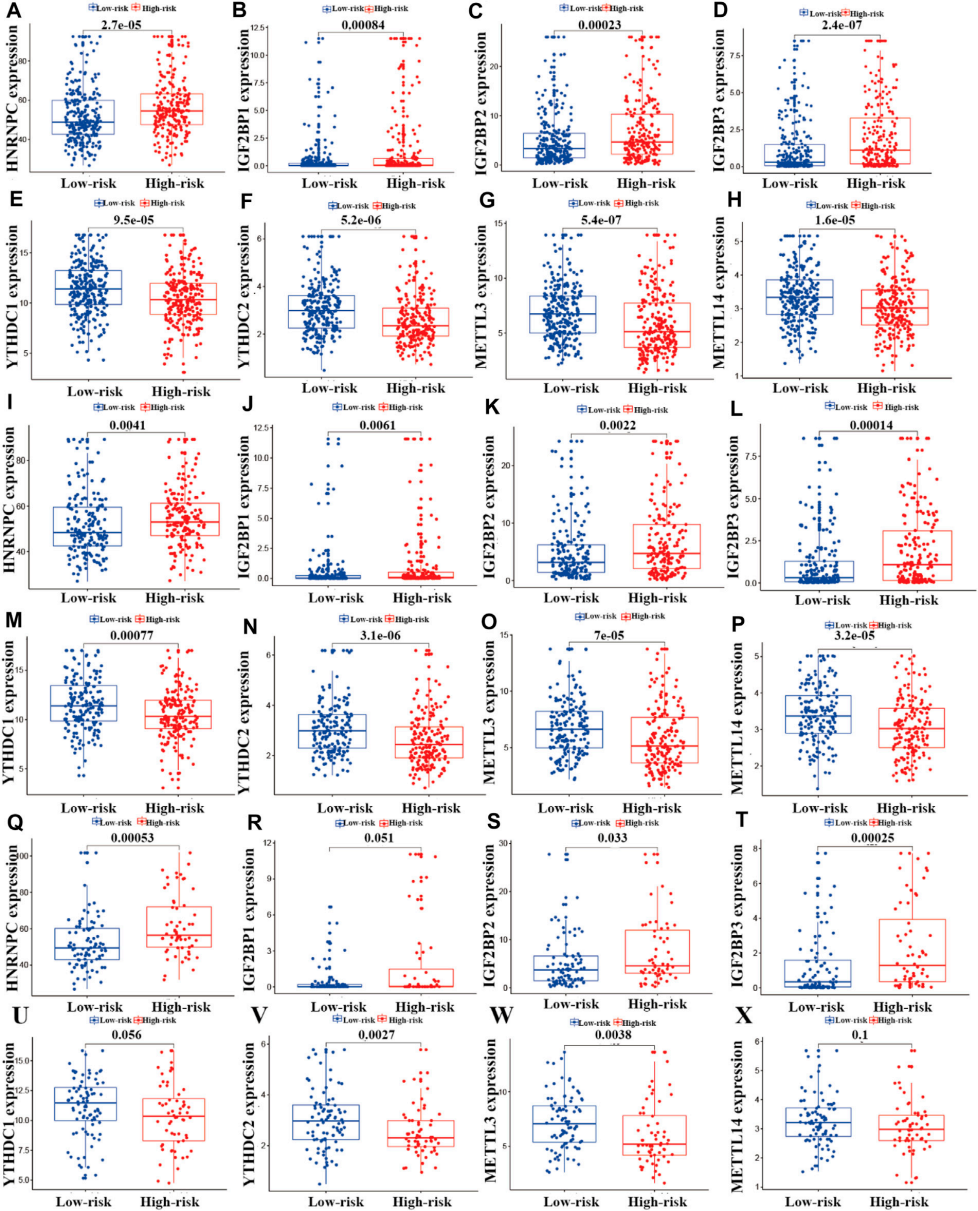
Correlation analysis of m^6^A regulators and the prognostic risk model. **(A–X)** Differential analysis of m^6^A regulators in the prognostic model for the high- and low-risk cohorts.

To investigate the relationships between m^6^A regulators and the risk model, we performed correlation analysis. As shown in Figure 8, eight m^6^A regulators (HNRNPC, YTHDC1, METTL3, IGF2BP1, METTL14, YTHDC2, IGF2BP2, and IGF2BP3) were significantly associated with the risk model (*p* < 0.05). In the entire LUAD patient cohort, HNRNPC, IGF2BP1, IGF2BP2, and IGF2BP3 had higher expression in the high-risk group than the low-risk group (*p* < 0.05; Figures 9A–D), whereas YTHDC1, YTHDC2, METTL3, and METTL14 had higher expression in the low-risk group (*p* < 0.05; Figures 9E–H). In the training cohort (*n* = 356), the expression of HNRNPC, IGF2BP1, IGF2BP2, and IGF2BP3 was significantly higher in the high-risk group than in the low-risk group (*p* < 0.05; Figure 8I-L), whereas the expression of YTHDC1, YTHDC2, METTL3, and METTL14 was significantly higher in the low-risk group (*p* < 0.05; Figures 9M–P)**.** In the testing cohort (*n* = 148), the expression levels of HNRNPC, IGF2BP2, and IGF2BP3 were higher in the high-risk group (*p* < 0.05; Figure 9Q, S, T), whereas IGF2BP1 and METTL14 showed no significant difference in expression between the two risk groups (*p* > 0.05; Figures 9R, X), and YTHDC1, YTHDC2, and METTL3 expression was higher in the low-risk group (*p* < 0.05; Figures 9U–W).

### Correlation Analysis Between Somatic Mutation Count and the Risk Model in the LUAD Cohort

**FIGURE 10:**
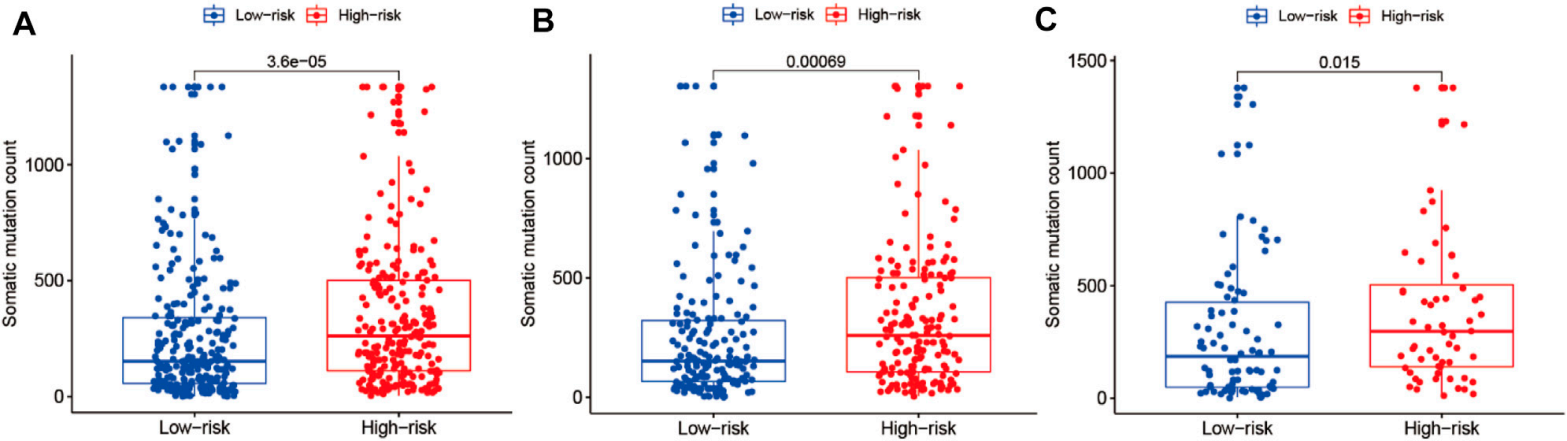
Correlation analysis of the prognostic risk model and somatic mutation counts of LUAD. **(A–C)** Differential analysis of somatic mutation counts in the prognostic model for the high- and low-risk cohorts.

We performed correlation analysis between somatic mutation count and the risk model. As shown in Figures 10A–C, the somatic mutation counts differed significantly between the high- and low-risk groups in the entire training and testing LUAD cohorts (*p* < 0.05). As shown in Figure 10, the somatic mutation counts were significantly higher in the high-risk groups than in the low-risk groups in all three cohorts (*p* < 0.05).

### Riskplot Analyses

**FIGURE 11:**
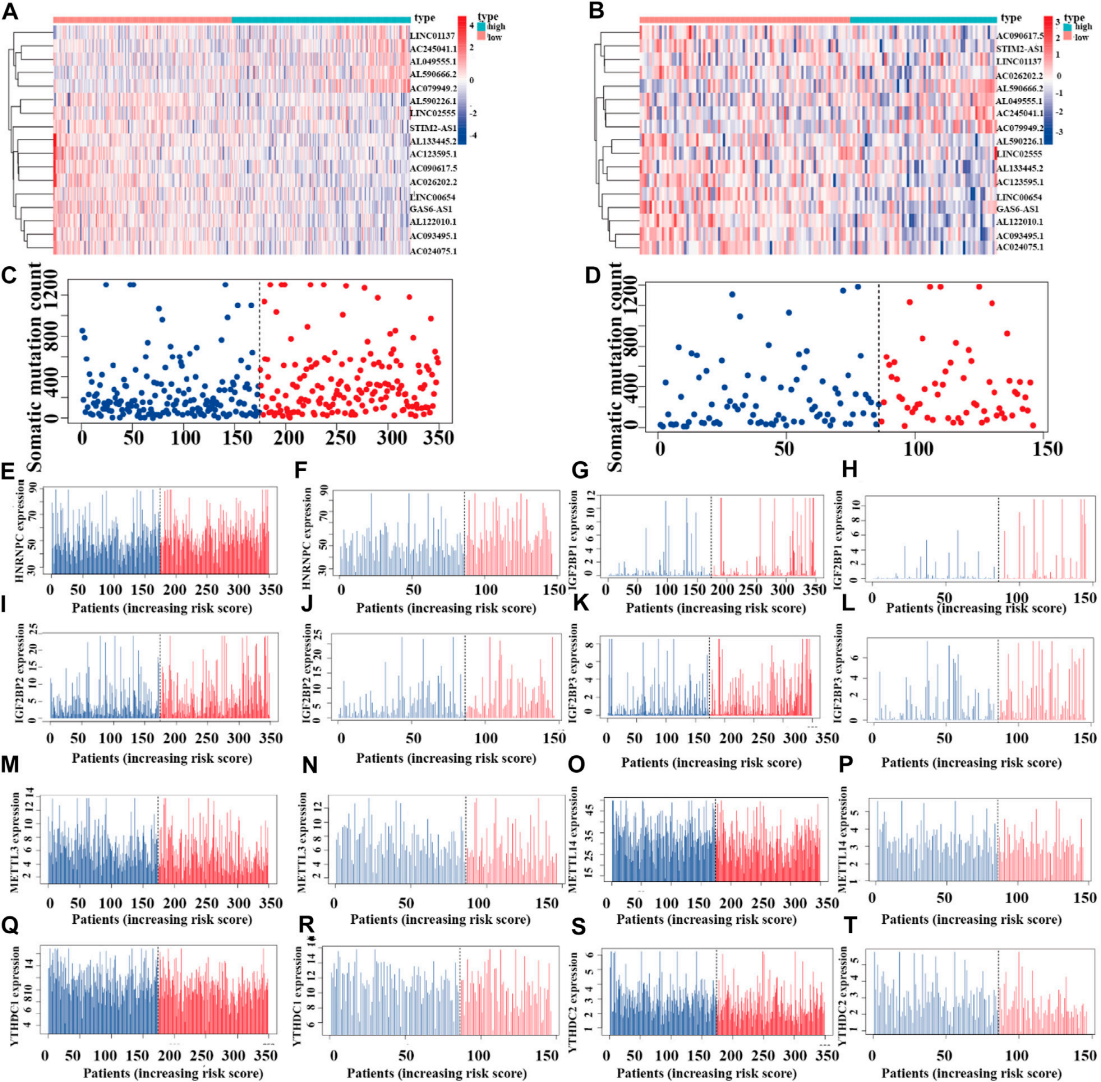
Riskplot analysis of m^6^A-related regulators **(A,C)** Riskplot heatmap and somatic mutation counts for the prognostic model in the training cohort of LUAD patients. **(B,D)** Riskplot heatmap and somatic mutation counts for the prognostic model in the testing cohort of LUAD patients. **(E,G,I,K,M,O,Q,S)** Differences in expression of HNRNPC, IGF2BP1, IGF2BP2, IGF2BP3, METTL3, METTL14, YTHDC1, and YTHDC2 in the high- and low-risk groups of the training cohort. **(F,H,J,L,N,P,R,T)** Differences in the expression of HNRNPC, IGF2BP1, IGF2BP2, IGF2BP3, METTL3, METTL14, YTHDC1, and YTHDC2 in the high- and low-risk groups of the testing cohort.

To reveal the relationships between the riskplot and expression and genomic instability of HNRNPC, IGF2BP1, METTL14, IGF2BP2, YTHDC1, IGF2BP2, YTHDC2, and METTL3, we performed riskplot analyses. In the training group, LUAD patient risk increased and the expression levels of LINC01137, AC245041.1, AL049555.1, AL590666.2, and AC079949.2 increased, whereas those of AL590226.1, LINC02555, STIM2-AS1, AL133445.2, AC123595.1, AC090617.5, AC026202.2, LINC00654, GAS6-AS1, AL122010.1, AC093495.1, and AC024075.1 decreased (Figure 11A). In the testing group of prognostic m^6^A-related lncRNA signature the heatmap was consistent with that in the training group; LINC01137, AC245041.1, AL049555.1, AL590666.2, AC079949.2 were high-risk factors, whereas AL590226.1, LINC02555, STIM2-AS1, AL133445.2, AC123595.1, AC090617.5, AC026202.2, LINC00654, GAS6-AS1, AL122010.1, AC093495.1, and AC024075.1 were low-risk factors (Figure 11B). In the training andtesting groups, LUAD patient risk increased and somatic mutation counts decreased (Figures 11C,D). There were differences in the expression of genomic instability of HNRNPC, IGF2BP1, IGF2BP2, IGF2BP3, METTL3, METTL14, YTHDC1, and YTHDC2 between high- and low-risk patients in the training set (Figures 11E,G,I,K, M, O, Q, S). There were also differences in the expression of genomic instability of HNRNPC, IGF2BP1, IGF2BP2, IGF2BP3, METTL3, METTL14, YTHDC1, and YTHDC2 between high- and low-risk patients in the testing set (Figures 11F,H,J, L, N, P, R, T).

### Prognostic Model Validation for Clinical Grouping

**FIGURE 12:**
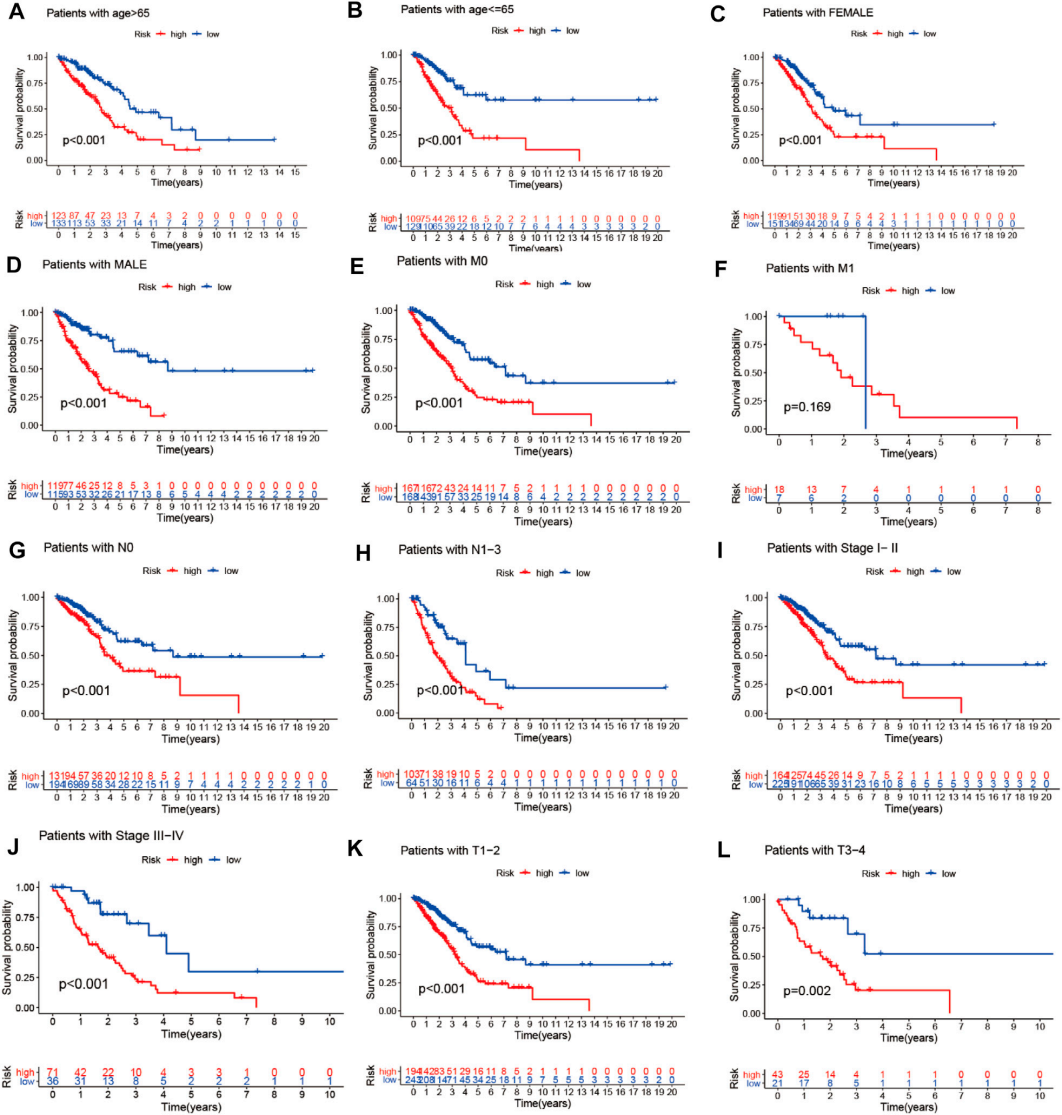
Prognostic model validation of clinical grouping. **(A–L)** Survival analysis of LUAD patients by age, gender, pathological M0/M1 stage, pathological T1–2/3–4 stage, pathological N0/N1–3 stage, and pathological stage I–II/III–IV in the high- and low-risk cohorts.

We combined the clinical data of 522 LUAD patients from TCGA with complete survival information and the risk file of the prognosis model for survival verification of clinical samples. Patients with LUAD in the high-risk cohort had worse prognosis than those in the low-risk cohort, regardless of whether they were older than 65 years or younger than 65 years (*p* < 0.001) (Figures 12A,B), or whether they were male or female (*p* < 0.001) (Figures 12C,D). Patients with pathological M0 stage in the high-risk cohort had poorer prognosis than those in the low-risk cohort (*p* < 0.001) (Figure 12E). However, there was no such significant difference with respect to pathological M1 stage between the high- and low-risk cohorts (*p* = 0.169) (Figure 12F). Among LUAD patients with pathological N0/N1-3 stage, the low-risk cohort had better prognosis than the high-risk cohort (*p* Figures 12G,H< 0.001) (Figures 12G,H). LUAD patients with pathological stages I–II/III–IV in the high-risk cohort had poorer prognosis than those in the low-risk cohort (*p* < 0.001) (Figures 12I,J). LUAD patients with pathological T1–2/3–4 stages in the high-risk cohort had poorer survival than those in the low-risk cohort (*p* < 0.05) (Figures 12K,L)

### Prognosis Validation of m^6^A-Related lncRNAs

We performed survival analysis of the 17 prognosis m^6^A-related lncRNAs (AL122010.1, STIM2-AS1, LINC00654, AL133445.2, GAS6-AS1, AC090617.5, AC093495.1, AC026202.2, AL590666.2, AC123585, AL590226.1, AC245041.1, LINC02555, AL049555.1, AC024075.1, AC079949.2, and LINC01137) from TCGA (https://portal.gdc.cancer.gov/) LUAD (lung adenocarcinoma) project in level 3 HTSeq-FPKM (fragments per kilobase per million) format RNA-seq data and survival information of 594 LUAD patients from TCGA database to verify the prognosis of the 17 lncRNAs. As shown in Supplementary Figure S1, patients with high expression levels of STIM2-AS1, LINC00654, AL133445.2, GAS6-AS1, AC090617.5, AC123595.1, and AC024075.1 had better prognosis than those with low expression levels, showing that these m^6^A-related lncRNAs are protective factors regarding the prognosis of LUAD patients, while the patients with high expression of AL590666.2, AL049555.1, and LINC01137 had poor prognosis than those with low expression, indicating that AL590666.2, AL049555.1, and LINC01137 are risk factors for predicting the survival of LUAD.

### Expression, Clinical Correlation, Immune Microenvironment, and Anticancer Drug Sensitivity Analyses of 17 Prognostic m^6^A-Related lncRNAs in LUAD

**FIGURE 13:**
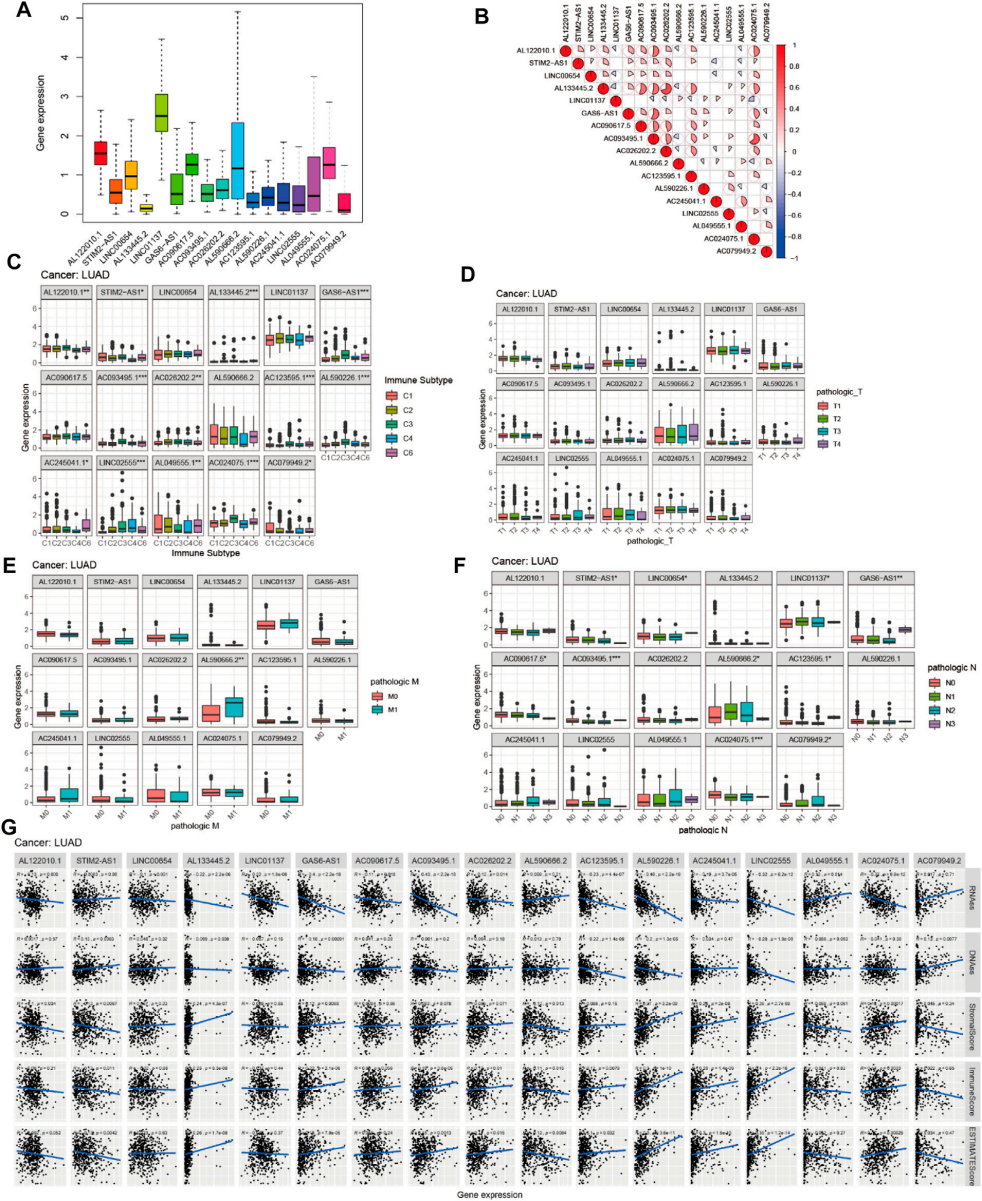
Expression, clinical correlation, and immune microenvironment analyses of prognostic m^6^A-related lncRNAs in LUAD. **(A)** Expression of 17 prognostic m^6^A-related lncRNAs in LUAD. **(B)** Correlation analysis of 17 prognostic m^6^A-related lncRNAs. **(C)** Boxplot of 17 prognostic m^6^A-related lncRNAs in six LUAD immune subtypes. **(D–F)** Boxplots of 17 prognostic m^6^A-related lncRNAs and clinical features in LUAD. **(G)** Correlations between the expression of 17 prognostic m^6^A-related lncRNAs and tumor stemness scores (based on RNA methylation and DNA methylation), immune score, estimate score, and stromal score in LUAD.

**FIGURE 14:**
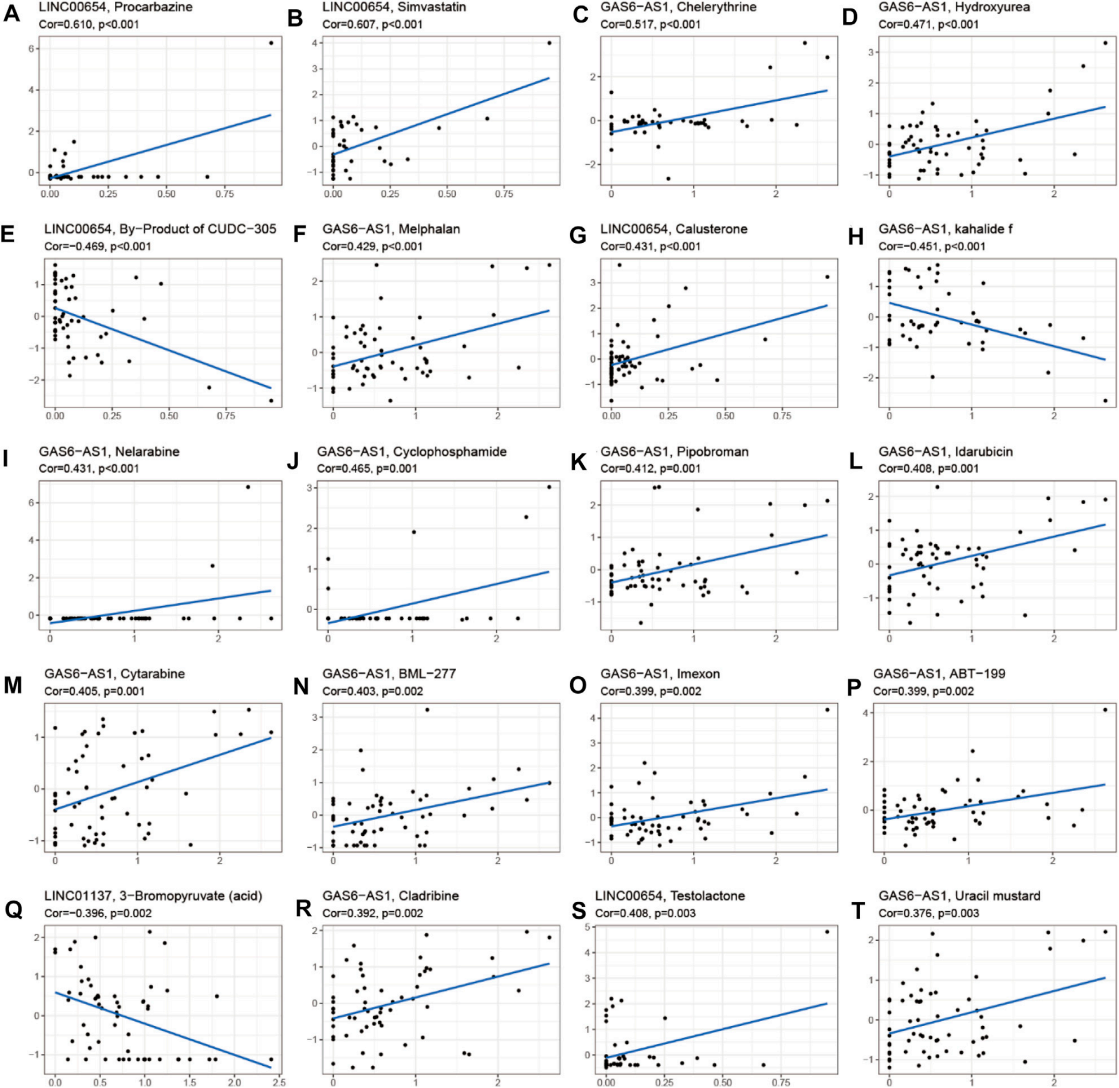
Correlation analysis between m^6^A-related lncRNAs and sensitivity to anticancer drugs in CellMiner. Top 20 statistically significant correlations between prognostic m^6^A-related lncRNAs and sensitivity to anticancer drugs.

To investigate the role of the 17 m^6^A-associated lncRNAs in LUAD, we downloaded the LUAD data from UCSC-Xena TCGA GDC to conduct differential expression, LUAD immune subtype, LUAD immune microenvironment, LUAD stemness score (DNAss and RNAss), and LUAD drug sensitivity analyses. As shown in Figure 13A, LINC01137 had higher expression levels than the other prognostic lncRNAs in LUAD. The expression levels of AC093495.1 and AC024075.1, and those of AL133445.2 and AC026202.2, were positively correlated with LUAD (Figure 13B). Expression levels of AL122010.1, STIM2-AS1, AL133445.2, GAS6-AS1, AC093495.1, AC026202.2, AC123595.1, AL590226.1, AC245041.1, LINC02555, AL049555.1, AC024075.1, and AC079949.2 were significantly different in immune subtypes C1 (wound healing), C4 (lymphocyte depleted), C3 (inflammatory), C2 (IFN-gamma dominant), and C6 (TGF-beta dominant) (Figure 13C). There was no statistically significant difference in the expression of the 17 prognostic m^6^A-related lncRNAs between pathological types T1/T2/T3/T4 (Figure 13D). However, the expression of AL590666.2 was significantly different in pathological stage M0/M1 of LUAD (*p* < 0.01) (Figure 13E). The expression levels of m^6^A-related lncRNAs STIM2-AS1, LINC00654, LINC01137, GAS6-AS1, AC090617.5, AC093495.1, AL590666.2, AC123595.1, AC024075.1, and AC079949.2 were significantly different in pathological stages N0/N1/N2/N3 (*p* < 0.05) (Figure 13F). As shown in Figure 13G, most of the m^6^A-related lncRNAs had negative correlations of their expression with RNAss and DNAss in LUAD, that is, higher expression of m6A-related lncRNAs, lower RNAss and DNAss stemness scores, weaker activity of LUAD stem cells, and greater degree of LUAD differentiation. The expression of LINC01137 was negatively correlated with RNAss in LUAD. The expression levels of most m^6^A-related lncRNAs were positively correlated with immune score, estimate score, and stromal score, which indicated that the content of immune and stromal cells was higher in LUAD, whereas tumor cell contents were lower in LUAD, indicating that most of the m^6^A-related lncRNAs were protective factors with respect to the survival of LUAD patients. As shown in Figure 14, LINC00654 expression was positively correlated with sensitivity to anticancer drugs procarbazine (*p* < 0.001), simvastatin (*p* < 0.001), testolactone (*p* = 0.003), and calusterone (*p* < 0.001) but negatively associated with sensitivity to anticancer drug byproduct of CUDC-305 (*p* < 0.001). The expression of GAS6-AS1 had a positive relationship with sensitivity to anticancer drugs chelerythrine (*p* < 0.001), hydroxyurea (*p* < 0.001), melphalan (*p* < 0.001), nelarabine (*p* < 0.001), cyclophosphamide (*p* = 0.001), pipobroman (*p* = 0.001), idarubicin (*p* = 0.001), cytarabine (*p* = 0.001), BML-277 (*p* = 0.002), imexon (*p* = 0.002), ABT-199 (*p* = 0.002), cladribine (*p* = 0.002), and uracil mustard (*p* = 0.003) but was negatively correlated with kahalidef sensitivity (*p* < 0.001). The expression of LINC01137 was negatively correlated with sensitivity to anticancer 3-bromopyruvate (acid) in LUAD (*p* = 0.002).

### Verification of Expression of 17 Prognostic m^6^A-Associated lncRNAs in LUAD

The expression levels of LINC01137, AL590666.2, AL049555.1, AC245041.1, LINC02555, AL590226.1, AL122010.1, AC093495.1, and AC090675.1 were significantly different between 57 paired LUAD and adjacent non-LUAD tissues (*p* < 0.001). The expressions of AC123595.1 and AC026202.2 in paired LUAD tissues were higher than those in paired non-LUAD tissues (*p* < 0.01). The expressions of AL133445.2 and AL123595.1 in paired LUAD tissues were lower than those in paired non-LUAD tissues (*p* < 0.01). The expressions of STIM2-AS1, LINC01137, AL590666.2, AL049555.1, AC079949.2, and AC026202.2 in unpaired LUAD tissues were higher than those in unpaired non-LUAD tissues (*p* < 0.001). The expressions of LINC02555, AL590226.1, AL133445.2, AL122010.1, AC245041.1, AC123595.1, AC093495.1, AC090617.5, and AC024075.1 in unpaired LUAD tissues were lower than those in unpaired non-LUAD tissues (*p* < 0.001). The expressions of 17 prognostic m^6^A-associated lncRNAs in paired LUAD, adjacent non-LUAD tissues, and unpaired LUAD and non-LUAD tissues are shown in Supplementary Figures S2, 3. The differential expression of 17 prognostic m^6^A-associated lncRNAs in LUAD and adjacent non-LUAD tissues is shown in Supplementary Table S3. Supplementary Table 3 showed that the log(fold change (FC)) of AL590226.1, AC245041.1, LINC02555, AC024075.1, AC090617.5, AC123595.1, and AC093495.1 was less than 0, which indicated that the expression of AL590226.1, AC245041.1, LINC02555, AC024075.1, AC090617.5, AC123595.1, and AC093495.1 was highly expressed in non-LUAD tissues compared with LUAD tissues (*p* < 0.05). The log FC of AL133445.2, AC079949.2, LINC00654, STIM2-AS1, GAS6-AS1, AC026202.2, LINC01137, AL049555.1, and AL590666.2 was greater than 0, which demonstrated that the expression of AL133445.2, AC079949.2, LINC00654, STIM2-AS1, AC026202.2, LINC01137, AL049555.1, and AL590666.2 was highly expressed in LUAD tissues than non-LUAD tissues (*p* < 0.05).

### Validation of 17 Prognostic m^6^A-Related lncRNAs in Human LUAD Cells and Normal Bronchial Epithelial Cells

To further evaluate whether the expression of 17 lncRNAs is significantly different between normal bronchial epithelial cells (Beas-2B) and LUAD cell lines (A549, H1299, and H1975), we performed the RT-qPCR. The results showed that the expression of LINC00654, AC090617.5, AC093495.1, AL590226.1, AC245041.1, AC123595.1, AL049555.1, AC079949.2, and LINC01137 was significantly higher in A549 than in Beas-2B (*p* < 0.05). The expression of AC079949.2, LINC01137, and AC024075.1 was significantly higher in H1299 than in Beas-2B (*p* < 0.05). The expression of AL122010.1, LINC00654, AC090617.5, GAS6-AS1, AC093495.1, AC026202.2, AL590226.1, AC245041.1, AL049555.1, AC079949.2, AL590666.2, LINC01137, and AC024075.1 was significantly different between Beas-2B and H1975 (*p* < 0.05). However, the expression of STIM2-AS1, AL133445.2, and LINC02555 had no significant difference between Beas-2B and A549, H1299, and H1975 (*p* > 0.05). The validation results are shown in Supplementary Figure S4.

### Validation of 22 Immune Cell Subtypes in High and Low Expression of 17 Prognostic m^6^A-Associated lncRNAs

To further validate the relationship between 17 prognostic m^6^A-associated lncRNAs and the immune microenvironment, we performed the CIBERSORT algorithm. The results showed that the fraction of naïve B cells was significantly different in the high- and low-expression groups of AL122010.1 and LINC00654 (*p* = 0.008, *p* = 0.031); the fraction of memory B cells was significantly different in the low- and high-expression groups of GAS6-AS1 (*p* = 0.017); the fraction of plasma cells was significantly different in the high- and low-expression groups of AC026202.2, AC090617.5, AC245041.1, AL049555.1, AL122010.1, LINC02555, and STIM2-AS1; the fraction of CD8 T cells was significantly different in the low- and high-expression groups of AC245041.1, AL049555.1, and LINC01137; the fraction of resting memory CD4 T cells was significantly different in the low- and high-expression groups of AC024075.1,AC093495.1, AL590226.1, GAS6-AS1, LINC00654, and LINC02555; the fraction of memory activated T cells was significantly different in the high- and low-expression groups of AC093495.1, AL049555.1, AL122010.1, AL590226.1, GAS6-AS1, and LINC01137; the fraction of follicular helper T cells was significantly different in the high- and low-expression groups of LINC01137 and AC024075.1; the fraction of regulatory T cells (Tregs) was significantly different in the high- and low-expression groups of AC090617.5, AC093495.1, AC245041.1, AL049555.1, GAS6-AS1, LINC01137, and LINC02555; the fraction of gamma delta T cells was significantly different in the high- and low-expression groups of AC026202.2, AL122010.1, and AL590666.2; the fraction of resting NK cells was significantly different in the high- and low-expression groups of LINC01137 and AL590226.1; the fraction of activated NK cells was significantly different in the high- and low-expression groups of AC123595.1, AL590666.2, and LINC01137; the fraction of monocytes was significantly different in the high- and low-expression groups of AC026202.2, GAS6-AS1, and LINC02555; the fraction of M0 macrophages was significantly different in the high- and low-expression groups of AC026202.2, AL590226.1, LINC01137, and LINC02555; the fraction of M1 macrophages was significantly different in the high- and low-expression groups of AC123595.1, AC245041.1, AL049555.1, LINC01137, and STIM2-AS1; the fraction of macrophages was significantly different in the high- and low-expression groups of AC026202.2, AC245041.1, AL122010.1, AL590226.1, LINC02555, and STIM2-AS1; the fraction of resting dendritic cells was significantly different in the low- and high-expression groups of AC093495.1, AL133445.2, AL590226.1, GAS6-AS1, LINC01137, and LINC02555; the fraction of activated dendritic cells was significantly different in the high- and low-expression groups of AC024075.1, AC093495.1, AL590666.2, LINC01137, and LINC02555; the fraction of resting mast cells was significantly different in the low- and high-expression groups of AC024075.1, AC093495.1, AL590226.1, GAS6-AS1, LINC01137, and LINC02555; the fraction of activated mast cells was significantly different in the high- and low-expression groups of AC093495.1, AL590226.1, and LINC02555; the fraction of eosinophils was significantly different in the low- and high-expression groups of AC245041.1; and the fraction of neutrophils was significantly different in the high- and low-expression groups of AC026202.2, AC079949.2, AC245041.1, AL590666.2, LINC01137, and LINC02555. The CIBERSORT immune microenvironment analysis result is shown in Supplementary Figure S5.

## Discussion

In this study, we first identified m^6^A-related lncRNAs by correlation analysis. Then, we identified differentially expressed genomic instability–associated m^6^A-related lncRNAs and constructed a ceRNA network and combined m^6^A-related lncRNA expression with somatic mutation profiles based on the tumor genome to explore the ability of the prognostic m^6^A-associated lncRNA signature to predict outcomes in LUAD. We also identified differences in the expression of immune checkpoint blockade CTLA4, HAVCR2, PDCD1, and 4-1 BB (TNFRSF9) in the GS-like and GU-like groups, which provided novel insight into the correlation relationship between immune checkpoint blockade and genomic instability of LUAD. Furthermore, we constructed an m^6^A-related lncRNA risk model and compared it with other reported lncRNA models, which revealed novel insights for predicting the outcome of LUAD. Eventually, pan-cancer analysis of prognostic m^6^A-related lncRNAs provided new prospects for identifying pan-cancer therapeutic targets. Previous studies have demonstrated the pivotal role of m^6^A modification of lncRNAs in cancers (Fazi and Fatica, 2019). Recently, a study demonstrated the role of oncogenic lncRNA THOR in m^6^A modification (Liu et al., 2020a). Additionally, the m^6^A-induced lncRNA RP11 was identified as a new predictive biomarker that triggers the dissemination of colorectal cancer metastasis through upregulation of ZEB1 (Wu et al., 2019). Some studies showed that the m^6^A RNA methyltransferase METTL3/14 could enhance response to anti–PD-1 treatment in pMMR-MSI-L melanoma and CRC (Wang et al., 2020a). However, the pivotal role of genomic instability of m^6^A-related lncRNAs in LUAD remained unclear.

In this study, we first identified genomic instability of the m^6^A-related lncRNA-associated ceRNA network, revealing novel insights related to new therapeutic targets involving RNA epigenetic mechanisms. We constructed a genomic instability–associated DElncRNA-mediated ceRNA network and found that four DElncRNAs (SRGAP3-AS2, AC110619.1, ATP13A4-AS1, and PSORS1C3) were significantly different between the GS-like group and GU-like groups. Recently, studies have revealed that SRGAP3-AS2 could play an important role in predicting the prognosis of LUAD (Yang et al., 2018; Jin et al., 2020). However, whether the expression of AC110619.1, ATP13A4-AS1, PSORS1C3, and SRGAP3-AS2 was associated with genomic instability was still unclear. We first constructed a ceRNA network of genomic instability–associated m^6^A-related lncRNAs, revealing novel insights for exploring new RNA epigenetic regulatory mechanisms in LUAD. Furthermore, we combined the expression of m^6^A regulators and immune checkpoint blockades CTLA4 and HAVCR2 with somatic mutations of LUAD, providing a new approach to exploring novel immune therapeutic targets in LUAD. Recently, a study showed that CTLA4 can serve as a prognostic biomarker for predicting the survival of LUAD (Wang et al., 2020b). Furthermore, an immunogenic gene signature associated with immune checkpoint blockade provided novel therapeutic targets for immunology in LUAD (Ahluwalia et al., 2021).

In our study, we identified a signature of 17 prognostic m^6^A-related lncRNAs based on all LUAD patients, a training group, and a testing group for predicting the outcomes of LUAD. Furthermore, we conducted a performance comparison analysis of our prognostic model and two recently published lncRNA signatures; the results indicated that our prognostic model of m^6^A-associated lncRNAs performed better in predicting the clinical outcomes of LUAD patients than the other two lncRNA signatures. In addition, validation using the GEO dataset GSE102287 confirmed that high expression of LINC01137 was associated with better prognosis of LUAD patients than low expression of LINC01137. Some studies have identified AL122010.1 as a predictor of survival of breast cancer patients (Li et al., 2020; Ma et al., 2020). The short-lived lncRNA LINC01137 serves as a useful indicator of chemical stress responses (Tani et al., 2019). Low expression of lncRNA GAS6-AS1 is a biomarker for predicting survival in NSCLC (Han et al., 2013). The expression of AC079949.2 is associated with clinical outcomes of patients with esophageal cancer (Liu et al., 2020b).

In our study, we identified the correlations between the expression of 17 prognostic m^6^A-related lncRNAs and the LUAD immune microenvironment, microenvironment, stemness scores, and immune subtype using the UCSC-Xena website. The immune microenvironment analysis showed that the 17 prognostic m^6^A-related lncRNAs were protective factors regarding clinical outcome on a pan-cancer basis. Recently, the m^6^A-related lncRNA LINC00958 was identified as a therapeutic target in hepatocellular carcinoma (Zuo et al., 2020). Other studies showed that ALKBH5 could promote the metastasis and invasion of gastric cancer by demethylating the expression of lncRNA NEAT1 (Zhang et al., 2019b) and that METTL14 acts as a prognostic biomarker in colorectal cancer by downregulating oncogenic lncRNA XIST (Yang et al., 2020). We constructed an m^6^A-related lncRNA prognostic risk model based on all LUAD patients, a training group, and a testing group from UCSC-Xena GDC TCGA-LUAD. Performance comparison analysis confirmed that the m^6^A-related lncRNA risk model could better predict clinical outcomes of LUAD patients than the previously published lncRNA signatures. Our study first verified the relationship between prognostic m^6^A-associated lncRNAs and the immune microenvironment based on the CIBERSORT algorithm, which provided a novel insight for revealing potential immune therapeutic targets in RNA epigenetics.

We first identified prognostic genomic instability of m^6^A-related lncRNAs in LUAD and constructed and validated a prognostic model based on the internal datasets (all TCGA-LUAD, training TCGA-LUAD, and testing TCGA-LUAD); furthermore, we validated the prognostic value of m^6^A-related lncRNA LINC01137 in the external dataset GSE102287 and analyzed the expression, clinical correlation, immune microenvironment, and anticancer drug sensitivity of the prognostic value of the 17 m^6^A-related lncRNAs in LUAD; the results provided novel insights for exploring new therapeutic targets in LUAD. Our study subjects were extracted from TCGA, the UCSC-Xena database, and the GEO database; it is not known whether our findings apply to other groups. We further validated the expression of prognostic m^6^A-related lncRNAs in normal bronchial epithelial (Beas-2B) and LUAD cells (A549, H1299, and H1975), and the results showed that the expression of AL122010.1, AC026202.2, AC123595.1, AL049555.1, AC079949.2, and LINC01137 was significantly higher in LUAD cells than normal bronchial epithelial cells (*p* < 0.05), which is consistent with TCGA database. However, the reason why the expression levels of the remaining lncRNAs are contrary to the results in the database may be related to ethnicity. In future, many molecular biology experiments will be performed, and many clinical samples will be collected to verify the 17 prognostic m^6^A-related lncRNAs.

In this study, we further explored the relationships between the expression levels of 17 prognostic m^6^A-associated lncRNAs and pathological N1-3/N0 and pathological M0/M1 stages. AL590666.2 was significantly different in pathological stages M0/M1, and 10 prognostic m^6^A-associated lncRNAs were differentially expressed in pathological N0/N1-3 stages. Our study first explored the association of the expression of m^6^A-associated lncRNAs with anticancer drug sensitivity using the CellMiner database, which provided novel insights regarding new therapeutic targets based on RNA epigenetics in LUAD.

## Conclusion

In conclusion, we identified the genomic instability of m^6^A-related lncRNAs and constructed a ceRNA network and then constructed and validated the Cox prediction model based on m^6^A-related lncRNAs using all TCGA-LUAD patients, a training group, and a testing group. Finally, clinical, prognostic, immune microenvironment, stemness scores, and anticancer drug sensitivity analyses of m^6^A-related lncRNAs in LUAD shed new light on potential therapeutic targets based on RNA epigenetics.

## Data Availability

The datasets presented in this study can be found in online repositories. The names of the repository/repositories and accession number(s) can be found in the article/Supplementary Material.

## References

[B1] Ahluwalia P. Ahluwalia M. Mondal A. K. Sahajpal N. Kota V. Rojiani M. V. (2021). Immunogenomic Gene Signature of Cell-Death Associated Genes with Prognostic Implications in Lung Cancer. Cancers 13, 155. 10.3390/cancers13010155 PMC7795632 33466402

[B2] Andor N. Maley C. C. Ji H. P. (2017). Genomic Instability in Cancer: Teetering on the Limit of Tolerance. Cancer Res. 77, 2179–2185. 10.1158/0008-5472.CAN-16-1553 28432052 PMC5413432

[B3] Bao S. Zhao H. Yuan J. Fan D. Zhang Z. Su J. (2020). Computational Identification of Mutator-Derived lncRNA Signatures of Genome Instability for Improving the Clinical Outcome of Cancers: a Case Study in Breast Cancer. Brief Bioinform 21, 1742–1755. 10.1093/bib/bbz118 31665214

[B4] Bray F. Ferlay J. Soerjomataram I. Siegel R. L. Torre L. A. Jemal A. (2018). Global Cancer Statistics 2018: GLOBOCAN Estimates of Incidence and Mortality Worldwide for 36 Cancers in 185 Countries. CA: A Cancer J. Clinicians 68, 394–424. 10.3322/caac.21492 30207593

[B5] Chu W. Zhang X. Qi L. Fu Y. Wang P. Zhao W. (2020). The EZH2-PHACTR2-AS1-Ribosome Axis Induces Genomic Instability and Promotes Growth and Metastasis in Breast Cancer. Cancer Res. 80, 2737–2750. 10.1158/0008-5472.can-19-3326 32312833

[B6] Chua M. L. K. Lo W. Pintilie M. Murgic J. Lalonde E. Bhandari V. (2017). A Prostate Cancer " Nimbosus ": Genomic Instability and SChLAP1 Dysregulation Underpin Aggression of Intraductal and Cribriform Subpathologies. Eur. Urol. 72, 665–674. 10.1016/j.eururo.2017.04.034 28511883

[B7] Duijf P. H. G. Nanayakkara D. Nones K. Srihari S. Kalimutho M. Khanna K. K. (2019). Mechanisms of Genomic Instability in Breast Cancer. Trends Mol. Med. 25, 595–611. 10.1016/j.molmed.2019.04.004 31078431

[B8] Fazi F. Fatica A. (2019). Interplay between N6-Methyladenosine (m6A) and Non-coding RNAs in Cell Development and Cancer. Front. Cel Dev. Biol. 7, 7. 10.3389/fcell.2019.00116 PMC6611489 31316981

[B9] Han L. Kong R. Yin D.-D. Zhang E.-B. Xu T.-P. De W. (2013). Low Expression of Long Noncoding RNA GAS6-AS1 Predicts a Poor Prognosis in Patients with NSCLC. Med. Oncol. 30, 694. 10.1007/s12032-013-0694-5 23979857

[B10] Hypoxic Tumors Share Genomic Instability, Cancer Discov..(2019); 9: 314. 10.1158/2159-8290.CD-NB2019-012 30723077

[B11] Jin D. Song Y. Chen Y. Zhang P. (2020). Identification of Three lncRNAs as Potential Predictive Biomarkers of Lung Adenocarcinoma. Biomed. Res. Int. 2020, 1–13. 10.1155/2020/7573689 PMC7053454 32149133

[B12] Jordan E. J. Kim H. R. Arcila M. E. Barron D. Chakravarty D. Gao J. (2017). Prospective Comprehensive Molecular Characterization of Lung Adenocarcinomas for Efficient Patient Matching to Approved and Emerging Therapies. Cancer Discov. 7, 596–609. 10.1158/2159-8290.CD-16-1337 28336552 PMC5482929

[B13] Kamburov A. Stelzl U. Lehrach H. Herwig R. (2013). The ConsensusPathDB Interaction Database: 2013 Update. Nucleic Acids Res. 41, D793–D800. 10.1093/nar/gks1055 23143270 PMC3531102

[B14] Lee S. Kopp F. Chang T.-C. Sataluri A. Chen B. Sivakumar S. (2016). Noncoding Rna Norad Regulates Genomic Stability by Sequestering Pumilio Proteins. Cell 164, 69–80. 10.1016/j.cell.2015.12.017 26724866 PMC4715682

[B15] Li X. Li Y. Yu X. Jin F. (2020). Identification and Validation of Stemness-Related lncRNA Prognostic Signature for Breast Cancer. J. Transl Med. 18, 331. 10.1186/s12967-020-02497-4 32867770 PMC7461324

[B16] Liu H. Xu Y. Yao B. Sui T. Lai L. Li Z. (2020). A Novel N6-Methyladenosine (m6A)-dependent Fate Decision for the lncRNA THOR. Cell Death Dis 11, 11. 10.1038/s41419-020-02833-y 32792482 PMC7426843

[B17] Liu H. Zhang Q. Lou Q. Zhang X. Cui Y. Wang P. (2020). Differential Analysis of lncRNA, miRNA and mRNA Expression Profiles and the Prognostic Value of lncRNA in Esophageal Cancer. Pathol. Oncol. Res. 26, 1029–1039. 10.1007/s12253-019-00655-8 30972633

[B18] Ma W. Zhao F. Yu X. Guan S. Suo H. Tao Z. (2020). Immune-related lncRNAs as Predictors of Survival in Breast Cancer: a Prognostic Signature. J. Transl Med. 18, 442. 10.1186/s12967-020-02522-6 33225954 PMC7681988

[B19] Munschauer M. Nguyen C. T. Sirokman K. Hartigan C. R. Hogstrom L. Engreitz J. M. (2018). The NORAD lncRNA Assembles a Topoisomerase Complex Critical for Genome Stability. Nature 561, 132–136. 10.1038/s41586-018-0453-z 30150775

[B20] Negrini S. Gorgoulis V. G. Halazonetis T. D. (2010). Genomic Instability - an Evolving Hallmark of Cancer. Nat. Rev. Mol. Cel Biol 11, 220–228. 10.1038/nrm2858 20177397

[B21] Ni W. Yao S. Zhou Y. Liu Y. Huang P. Zhou A. (2019). Long Noncoding RNA GAS5 Inhibits Progression of Colorectal Cancer by Interacting with and Triggering YAP Phosphorylation and Degradation and Is Negatively Regulated by the m6A Reader YTHDF3. Mol. Cancer 18, 143. 10.1186/s12943-019-1079-y 31619268 PMC6794841

[B22] Petti E. Buemi V. Zappone A. Schillaci O. Broccia P. V. Dinami R. (2019). SFPQ and NONO Suppress RNA:DNA-hybrid-related Telomere Instability. Nat. Commun. 10, 1001. 10.1038/s41467-019-08863-1 30824709 PMC6397292

[B23] Sahin I. H. Lowery M. A. Stadler Z. K. Salo-Mullen E. Iacobuzio-Donahue C. A. Kelsen D. P. (2016). Genomic Instability in Pancreatic Adenocarcinoma: a New Step towards Precision Medicine and Novel Therapeutic Approaches. Expert Rev. Gastroenterol. Hepatol. 10, 1–13. 10.1586/17474124.2016.1153424 26881472 PMC4988832

[B24] Seton-Rogers S. (2018). The Sting of Metastasis. Nat. Rev. Cancer 18, 137. 10.1038/nrc.2018.16 29467525

[B25] Sheffer M. Bacolod M. D. Zuk O. Giardina S. F. Pincas H. Barany F. (2009). Association of Survival and Disease Progression with Chromosomal Instability: a Genomic Exploration of Colorectal Cancer. Pnas 106, 7131–7136. 10.1073/pnas.0902232106 19359472 PMC2678450

[B26] Shukla S. Evans J. R. Malik R. Feng F. Y. Dhanasekaran S. M. Cao X. (2017). Development of a RNA-Seq Based Prognostic Signature in Lung Adenocarcinoma JNCI J. Natl. Cancer Inst. 109, djw200. 10.1093/jnci/djw200 PMC5051943 27707839

[B27] Sun J. Zhang Z. Bao S. Yan C. Hou P. Wu N. (2020). Identification of Tumor Immune Infiltration-Associated lncRNAs for Improving Prognosis and Immunotherapy Response of Patients with Non-small Cell Lung Cancer. J. Immunother. Cancer 8, 1–12. 10.1136/10.1136/jitc-2019-000110 PMC7057423 32041817

[B28] Tani H. Numajiri A. Aoki M. Umemura T. Nakazato T. (2019). Short-lived Long Noncoding RNAs as Surrogate Indicators for Chemical Stress in HepG2 Cells and Their Degradation by Nuclear RNases. Sci. Rep. 9, 20299. 10.1038/s41598-019-56869-y 31889167 PMC6937343

[B29] Torre L. A. Bray F. Siegel R. L. Ferlay J. Lortet-Tieulent J. Jemal A. (2015). Global Cancer Statistics, 2012. CA: A Cancer J. Clinicians 65, 87–108. 10.3322/caac.21262 25651787

[B30] Tracy K. M. Tye C. E. Ghule P. N. Malaby H. L. H. Stumpff J. Stein J. L. (2018). Mitotically-Associated lncRNA (MANCR) Affects Genomic Stability and Cell Division in Aggressive Breast Cancer. Mol. Cancer Res. 16, 587–598. 10.1158/1541-7786.MCR-17-0548 29378907 PMC5882506

[B31] Tu Z. Wu L. Wang P. Hu Q. Tao C. Li K. (2020). N6-Methylandenosine-Related lncRNAs Are Potential Biomarkers for Predicting the Overall Survival of Lower-Grade Glioma Patients. Front. Cel Dev. Biol. 8, 642. 10.3389/fcell.2020.00642 PMC7390977 32793593

[B32] Wang L. Hui H. Agrawal K. Kang Y. Li N. Tang R. (2020). M 6 A Rna Methyltransferases Mettl3/14 Regulate Immune Responses To Anti‐Pd‐1 Therapya Rna Methyltransferases Mettl3/14 Regulate Immune Responses To Anti-Pd-1 Therapy. EMBO J. 39, e104514. 10.15252/embj.2020104514 32964498 PMC7560214

[B33] Wang L. Luo X. Cheng C. Amos C. I. Cai G. Xiao F. (2020). A Gene Expression-Based Immune Signature for Lung Adenocarcinoma Prognosis. Cancer Immunol. Immunother. 69, 1881–1890. 10.1007/s00262-020-02595-8 32372138 PMC11027606

[B34] Wang Y. Lu J.-H. Wu Q.-N. Jin Y. Wang D.-S. Chen Y.-X. (2019). Lncrna Linris Stabilizes Igf2bp2 And Promotes The Aerobic Glycolysis In Colorectal Cancer. Mol. Cancer 18, 174. 10.1186/s12943-019-1105-0 31791342 PMC6886219

[B35] Wu Y. Yang X. Chen Z. Tian L. Jiang G. Chen F. (2019). m6A-induced lncRNA RP11 Triggers the Dissemination of Colorectal Cancer Cells via Upregulation of Zeb1. Mol. Cancer 18, 87. 10.1186/s12943-019-1014-2 30979372 PMC6461827

[B36] Yang X. Zhang S. He C. Xue P. Zhang L. He Z. (2020). METTL14 Suppresses Proliferation and Metastasis of Colorectal Cancer by Down-Regulating Oncogenic Long Non-coding RNA XIST. Mol. Cancer 19, 46. 10.1186/s12943-020-1146-4 32111213 PMC7047419

[B37] Yang Z. Li H. Wang Z. Yang Y. Niu J. Liu Y. (2018). Microarray Expression Profile of Long Non-coding RNAs in Human Lung Adenocarcinoma. Thorac. Cancer 9, 1312–1322. 10.1111/1759-7714.12845 30151992 PMC6166069

[B38] Zhang C. Zhang M. Ge S. Huang W. Lin X. Gao J. (2019). Reduced m6A Modification Predicts Malignant Phenotypes and Augmented Wnt/PI3K‐Akt Signaling in Gastric Cancer. Cancer Med. 8, 4766–4781. 10.1002/cam4.2360 31243897 PMC6712480

[B39] Zhang J. Guo S. Piao H.-y. Wang Y. Wu Y. Meng X.-y. (2019). ALKBH5 Promotes Invasion and Metastasis of Gastric Cancer by Decreasing Methylation of the lncRNA NEAT1. J. Physiol. Biochem. 75, 379–389. 10.1007/s13105-019-00690-8 31290116 PMC6728298

[B40] Zhang L. Luo Y. Cheng T. Chen J. Yang H. Wen X. (2021). Development and Validation of a Prognostic N6-Methyladenosine-Related Immune Gene Signature for Lung Adenocarcinoma. Pgpm Vol 14, 1549–1563. 10.2147/PGPM.S332683 PMC8643173 34876833

[B41] Zhao Y. Varn F. S. Cai G. Xiao F. Amos C. I. Cheng C. (2018). A P53-Deficiency Gene Signature Predicts Recurrence Risk of Patients with Early-Stage Lung Adenocarcinoma. Cancer Epidemiol. Biomarkers Prev. 27, 86–95. 10.1158/1055-9965.EPI-17-0478 29141854 PMC5839302

[B42] Zuo X. Chen Z. Gao W. Zhang Y. Wang J. Wang J. (2020). M6A-mediated Upregulation of LINC00958 Increases Lipogenesis and Acts as a Nanotherapeutic Target in Hepatocellular Carcinoma. J. Hematol. Oncol. 13, 5. 10.1186/s13045-019-0839-x 31915027 PMC6951025

