# Supplementary material for: Prognostic Value of Genomic Instability of m6A-Related lncRNAs in Lung Adenocarcinoma
Source: Front Cell Dev Biol. 2022 Mar 3;10:707405. doi: 10.3389/fcell.2022.707405 (PMC8928224; doi:10.3389/fcell.2022.707405)
Supplement: Supplementary file 2 [file Table1.docx]

Primer Sequence (5'->3')

AL122010.1-Forward primer CCACTAAGGCAGGTGTGGAG

AL122010.1-Reverse primer TGGTGAGGTGGGGATCTAGG

STIM2-AS1- Forward primer CCCATCGACTCTGTGCTGTA

STIM2-AS1- Reverse primer TCCAGGTTGGCGAAAACAAG

LINC00654- Forward primer TGAACTCCAGCCATGCCATT

LINC00654- Reverse primer CATTTTGCTTCCACGGCTCC

AL133445.2-Forward primer ATCCTACTGTGGTGTTGCGG

AL133445.2- Reverse primer GGCACTATCGCGTCTGTCAA

AC090617.5- Forward primer TGGGCTGGAGTATTTGTGCT

AC090617.5-Reverse primer GCCCTCAGACATAGTTGCCA

GAS6-AS1- Forward primer TTTGCCTGGAATGCCTTTCG

GAS6-AS1- Reverse primer TGTGGTTCCGTCCTTGCATC

AC026202.2- Forward primer AACAACTCCAACGGTCTCGG

AC026202.2- Reverse primer TATGCACTCCATTGGTCGGC

AC123595.1- Forward primer TCACTTCCGTTGTACCCAGC

AC123595.1- Reverse primer GCACACAACGATGCCAAGTT

AC093495.1- Forward primer CCTTTCCACCACACACGGT

AC093495.1- Reverse primer CAGGTCATCTGCTGGGATCT

AC123595.1- Forward primer TCACTTCCGTTGTACCCAGC

AC123595.1- Reverse primer GCACACAACGATGCCAAGTT

AL590226.1-Forward primer CACTCCACTCTCTTGGGCTCC

AL590226.1- Reverse primer TTGATGAGGGTTCTCTGCGTC

AC245041.1- Forward primer CCCTTCCAAGGAGGACACAC

AC245041.1- Reverse primer CAGGTGTCCAATAGCTGCCT

AL049555.1-Forward primer AAAGCCCAGAACCCACAGTT

AL049555.1- Reverse primer TTCAGAACTGCCTAATGGCTCA

AC024075.1- Forward primer AGAACAGCAGCAGTGCGAT

AC024075.1- Reverse primer CCTTGCTAATGACAGCTTCGC

AC079949.2- Forward primer CACTACCCGAATCTCAGTTGCT

AC079949.2- Reverse primer GGACCAATCCCTCATCCTGG

LINC01137- Forward primer AGGTAAAGCCGCGAACACTC

LINC01137- Reverse primer GCCGTACGTAGCTTCATCAG

AL590666.2- Forward primer TCAGGGACAGGGCAGTATTC

AL590666.2- Reverse primer CAACTCTCCCCAAGAGCGG

homo GAPDH- Forward primer GCACCGTCAAGGCTGAGAAC

homo GAPDH- Reverse primer TGGTGAAGACGCCAGTGGA

**Supplementary Table S1 The primer sequences of GAPDH and 17 prognostic m^6^A-related lncRNAs.**
