# Supplementary figures and images for: Prognostic Value of Genomic Instability of m6A-Related lncRNAs in Lung Adenocarcinoma
Source: Front Cell Dev Biol. 2022 Mar 3;10:707405. doi: 10.3389/fcell.2022.707405 (PMC8928224; doi:10.3389/fcell.2022.707405)

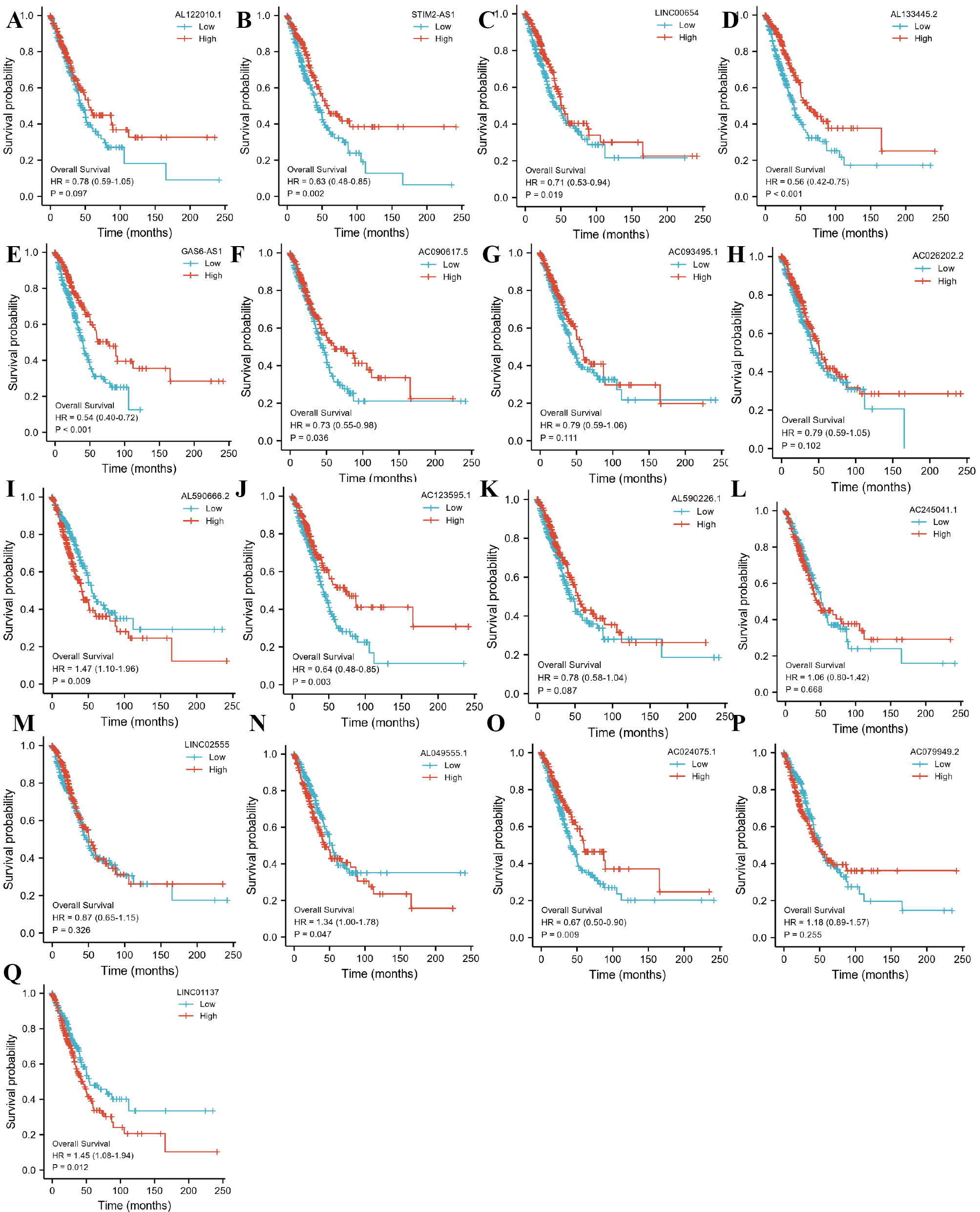

Supplement: Supplementary file 3 [file Image1.tif]
