# Supplementary material for: Prognostic Value of Genomic Instability of m6A-Related lncRNAs in Lung Adenocarcinoma
Source: Front Cell Dev Biol. 2022 Mar 3;10:707405. doi: 10.3389/fcell.2022.707405 (PMC8928224; doi:10.3389/fcell.2022.707405)
Supplement: Supplementary file 4 [file Table3.docx]

| LncRNA | logFC | *P*-value |
| --- | --- | --- |
| AL590226.1 | -2.01506 | 2.37E-33 |
| AC245041.1 | -1.54752 | 3.27E-24 |
| LINC02555 | -1.33283 | 8.05E-25 |
| AC024075.1 | -0.42079 | 2.19E-06 |
| AC090617.5 | -0.41628 | 3.73E-13 |
| AC123595.1 | -0.24098 | 1.29E-05 |
| AC093495.1 | -0.23788 | 1.05E-06 |
| AL122010.1 | -0.08383 | 0.101078 |
| AL133445.2 | 0.037233 | 0.001051 |
| AC079949.2 | 0.172501 | 0.003063 |
| LINC00654 | 0.17537 | 0.013124 |
| STIM2-AS1 | 0.188725 | 0.00555 |
| GAS6-AS1 | 0.20677 | 0.366156 |
| AC026202.2 | 0.243941 | 2.56E-05 |
| LINC01137 | 0.446514 | 1.57E-07 |
| AL049555.1 | 0.7 | 9.11E-06 |
| AL590666.2 | 1.172757 | 1.54E-18 |

**Supplementary Table S3.** **The differential expression of prognostic m^6^A related lncRNAs in LUAD and non-LUAD tissues.**
