# Supplementary material for: Prognostic Value of Genomic Instability of m6A-Related lncRNAs in Lung Adenocarcinoma
Source: Front Cell Dev Biol. 2022 Mar 3;10:707405. doi: 10.3389/fcell.2022.707405 (PMC8928224; doi:10.3389/fcell.2022.707405)
Supplement: Supplementary file 5 [file DataSheet1.docx]

**FIGURE LEGENDS**

**Supplementary Figure S1 Survival analysis of m6A-related lncRNAs in LUAD patients.** Red and blue line indicate LUAD patients with high and low expression of m6A-related lncRNAs.

**Supplementary Figure S2 Paired sample scatter line chart of expression of 17 prognostic m6A-related lncRNAs in paired LUAD tissues and adjacent non-LUAD tissues in TCGA.** Red dots indicate paired LUAD tissues. Blue dots indicate paired adjacent non-LUAD tissues. * indicated P < 0.05, ** indicated P < 0.01, *** indicated P < 0.001.

**Supplementary Figure S3 Boxplot of expression of 17 prognostic m6A-related lncRNAs in unpaired LUAD and non-LUAD tissues in TCGA.** Red and blue boxplot indicate LUAD and non-LUAD tissues, respectively. * indicated P < 0.05, ** indicated P < 0.01, *** indicated P < 0.001.

**Supplementary Figure S4 Validation of 17 prognostic m6A related lncRNAs in normal bronchial epithelial cell and LUAD cells.** * Indicated P < 0.05, ** indicated P < 0.01, *** indicated P < 0.001, **** indicated P < 0.001.

**Supplementary Figure S5 Violin of 22 immune cells fraction in prognostic m6A-related lncRNAs.** Red and green violin indicates high and low expression of lncRNAs, respectively.
